# Global prevalence of drug-resistant tuberculosis: a systematic review and meta-analysis

**DOI:** 10.1186/s40249-023-01107-x

**Published:** 2023-05-25

**Authors:** Nader Salari, Amir Hossein Kanjoori, Amin Hosseinian-Far, Razie Hasheminezhad, Kamran Mansouri, Masoud Mohammadi

**Affiliations:** 1 Department of Biostatistics, School of Health, Kermanshah University of Medical Sciences, Kermanshah, Iran; 2 Sleep Disorders Research Center, Kermanshah University of Medical Sciences, Kermanshah, Iran; 3 Student Research Committee, Kermanshah University of Medical Sciences, Kermanshah, Iran; 4 Department of Business Systems & Operations, University of Northampton, Northampton, UK; 5 Medical Biology Research Center, Kermanshah University of Medical Sciences, Kermanshah, Iran; 6 Cellular and Molecular Research Center, Gerash University of Medical Sciences, Gerash, Iran

**Keywords:** Prevalence, Drug-resistant tuberculosis, Burden, Outbreak, TB

## Abstract

**Background:**

Tuberculosis is a bacterial infectious disease, which affects different parts of a human body, mainly lungs and can lead to the patient’s death. The aim of this study is to investigate the global prevalence of drug-resistant tuberculosis using a systematic review and meta-analysis.

**Methods:**

In this study, the PubMed, Scopus, Web of Science, Embase, ScienceDirect and Google Scholar repositories were systematically searched to find studies reporting the global prevalence of drug-resistant tuberculosis. The search did not entail a lower time limit, and articles published up until August 2022 were considered. Random effects model was used to perform the analysis. The heterogeneity of the studies was examined with the *I*^*2*^ test. Data analysis was conducted within the Comprehensive Meta-Analysis software.

**Results:**

In the review of 148 studies with a sample size of 318,430 people, the *I*^*2*^ index showed high heterogeneity (*I*^*2*^ = 99.6), and accordingly random effects method was used to analyze the results. Publication bias was also examined using the Begg and Mazumdar correlation test which indicated the existence of publication bias in the studies (*P* = 0.008). According to our meta-analysis, the global pooled prevalence of multi-drug resistant TB is 11.6% (95% *CI*: 9.1–14.5%).

**Conclusions:**

The global prevalence of drug-resistant tuberculosis was found to be very high, thus health authorities should consider ways to control and manage the disease to prevent a wider spread of tuberculosis and potentially subsequent deaths.

**Supplementary Information:**

The online version contains supplementary material available at 10.1186/s40249-023-01107-x.

## Background

Tuberculosis (TB) is one of the most common infectious diseases, which is the main cause of widespread mortality, especially among people living with HIV (PLHIV) [1, 2]. The disease is caused by a type of bacteria called Mycobacterium TB [3]. Different types of TB are multi drug-resistant (MDR), pre-extensively drug-resistant (Pre-XDR), and extensively drug-resistant (XDR) [4]. TB usually affects the lungs, however it can also affect other parts of the body, such as the kidneys and the brain [4].

There were an estimated 450,000 incident cases of MDR in 2021, up 3.1% from 437,000 in 2020, three countries accounted for 42% of global cases in 202: India (26%), the Russian Federation (8.5%), and Pakistan (7.9%) [5]. A study by Baya et al., reported that the average age of patients was 39.31 ± 14.64 years, whilst 62.6% of patients were less than 40 years old. Patients were predominantly male 76.2%, and 77.1% were married [6]. Additionally, the prevalence of latent MDR TB has been reported in some countries of Eastern Europe and Central Asia, such as China (6 million people), India (4 million people), and Russia (1.8 million people) [4–6].

According to the existing literature, risk factors of tuberculosis include demographic characteristics such as gender, age, place of residence, education, marital status, bad habits such as alcohol abuse and smoking, and concomitant infections including diabetes mellitus, HIV, Acid-Fast Bacilli (AFB) smear, pulmonary space, history of tuberculosis, and history of anti-tuberculosis treatment are significant risk factors for MDR TB [7].

TB often impacts patients with other diseases such as diabetes, HIV, and chronic obstructive pulmonary disease (COPD) [7]. Cough or fever for > 2 weeks, weight loss, or hemoptysis are among the symptoms of TB which are also associated with lack of health insurance, tuberculin skin test, diagnosis through a process not entailing screening, and ethnicities other than Asian [8]. Complications of this disease include bronchial stenosis, severe airway obstruction, pneumonia, and hemoptysis, the most common of which is liver damage [9, 10]. Vocal cord paralysis, associated with laryngeal TB, can also be found among the patients [10]. To treat TB, a combination of isoniazid, rifampin, ethambutol, and pyrazinamide, followed by a combination of isoniazid and rifampin are used [9].

Several studies have been conducted on the prevalence of drug-resistant tuberculosis worldwide. These studies have reported different rates, yet their reported results are heterogeneous and are not aligned. The aim of this systematic review and meta-analysis is to pool the reported results of the existing studies and offer a scientifically consistent prevalence for drug resistant TB. The findings of our study can provide useful insights to health policymakers to devised appropriate interventions, with a view to reducing the subsequent complications from the disease.

## Methods

This systematic review and meta-analysis was conducted in accordance with the Preferred Reporting Items for Systematic Reviews and Meta-Analyses (PRISMA) guidelines. The keywords of prevalence, drug-resistant tuberculosis, burden, outbreak and their combination using the (AND) and (OR) operators, were used to search the PubMed, Google Scholar, Science Direct, Embase, Scopus and Web of Science databases. The search was conducted with no lower time limit and until August 2022. The reference lists within the identified studies were also manually searched to ensure the comprehensive of the collected articles. The information of the identified studies was transferred into the EndNote reference management software, and studies that had reported the prevalence of drug-resistant tuberculosis by continent and were satisfying the inclusion criteria, were selected for final analysis.

### Inclusion and exclusion criteria

The following criteria were used to keep an identified study in the systematic review and for meta-analysis: Studies that reported the prevalence of drug-resistant tuberculosis (including cross-sectional, case–control, and cohort studies), Studies with their full-text available, Studies that provided sufficient data (sample size, prevalence), Studies written and published in English. In contrary, the following criteria resulted in excluding identified articles: Case report studies, case series studies, duplicate studies and meta-analysis studies.

### Study selection

Similarly, selection of studies was conducted in accordance with the PRISMA guidelines. Initially, articles that were duplicates in different databases were excluded, and only one copy was retained. Subsequently, the initial screening of articles was conducted through reviewing the titles and abstracts, and irrelevant articles were omitted based on the inclusion and exclusion criteria. Then their full text of articles was reviewed in line with the inclusion and exclusion criteria, and at this stage further irrelevant studies were removed. To avoid any potential bias, all the steps of reviews and data extraction were conducted by two reviewers independently. In cases where there was a difference of opinion between two reviewers, the review of the article was finalized by a third reviewer.

### Quality evaluation

To evaluate the quality of articles, a checklist appropriate to observational studies was selected. The Strengthening the Reporting of Observational Studies in Epidemiology checklist (STROBE) consists of six scales including: title, abstract, introduction, methods, results, and discussion. In total, this instruction consists of 32 subscales. These 32 subscales denote different methodological aspects of the study, i.e., title, statement of the problem, study objectives, type of study, statistical population of the study, sampling method, determining the appropriate sample size, definition of variables and procedures, study data collection tools, statistical analysis methods and findings. Consider that the fulfilment of each of the subscales award a point, and based on this, articles with scores of 16 and above were considered to be of medium and high methodological quality articles respectively. Articles with a score below 16 were considered to be of poor quality and were therefore excluded from our study.

### Data extraction

Data extraction was completed by two researchers using a different pre-prepared checklist. This checklist included: first author's name, year of publication, study location, sample size, age group of men and women, global prevalence of drug-resistant tuberculosis, and research instruments.

### Statistical analysis

The extracted information were structured and were inputted into Comprehensive Meta-Analysis software (Version 2, Biostat, Inc., 14 North Dean Street, Englewood, NJ 07631 USA). The heterogeneity of the studies was then assessed using the *I*^*2*^ test. In order to check the publication bias, the Begg’s test was used at a significance level of 0.1, and associated Funnel plots were drawn.

## Results

**Fig. 1:**
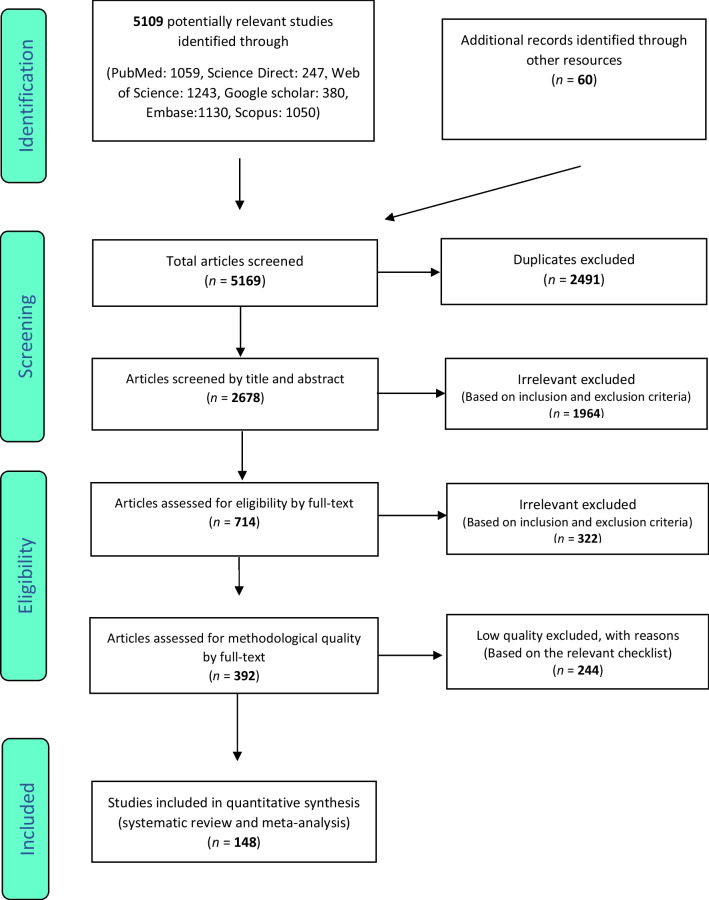
PRISMA flow diagram for study selection

Following the initial search, 5109 articles were identified from the databases. An additional 60 related articles were also included following manual searches. Information of all identified articles were then transferred into the EndNote reference management software. Throughout the PRISMA’s identification stage, 2491 articles were excluded due to being repeated in various databases, and only one copy was retained. In the screening stage, the title and abstract of the studies were reviewed and 1964 further articles were excluded based on the inclusion and exclusion criteria. In the eligibility evaluation phase, 323 articles were omitted, after examination of the full text of the articles. As part of quality evaluation, and through the evaluation of the full text of the articles and based on the scores obtained from the STROBE checklist, studies with poor methodological quality were removed, and finally 148 studies were kept for analysis. All included studies were cross-sectional and most of the reviewed studies were conducted in Africa (continent). The information related to the 148 included studies is presented in Fig. 1 and Additional file 1: Tables S1 to S6 [11–179].

### Multi drug-resistant TB

In the review of 148 studies that had studied multi drug resistant TB (sample size of 318,430 people), the *I*^*2*^ test showed a high heterogeneity (*I*^*2*^ = 99.6), and accordingly, random effects method was used to analyze the results. Considering the meta-analysis, the global pooled prevalence of multi-drug resistant TB was found to be 11.6% (95% *CI*: 9.1–14.5%). Test of publication bias in the studies through the Begg and Mazumdar correlation test showed the existence of publication bias among the studies (*P* = 0.008) (Table 1) (Figs. S1, S2 in Additional file 2).

**Table 1:** Meta-analysis of the prevalence of types of tuberculosis drug resistance

Type	*N*	Sample size	*I*^*2*^	Begg and Mazumdar correlation test	Meta-analysis of Prevalence (95% *CI*)
Multi drug-resistant TB	148	318,430	99.6	*P* = 0.008	11.6% (9.1–14.5%)
Isoniazid resistant TB	98	102,260	99.03	*P* = 0.02	15.7% (13.7–17.9%)
Rifampin resistant TB	109	215,660	98.9	*P* = 0.00045	9.4% (7.8–11.2%)
Single drug resistant TB	35	45,147	98.5	*P* = 0.139	11.8% (9.2–15.2%)
Extensive drug resistant TB	56	350,420	98.8	*P* = 0.938	2.5% (2–3%)

TB: Tuberculosis, *CI*: Confidence interval

### Isoniazid resistant TB

In 98 studies with a focus on Isoniazid resistant TB (sample size of 102,260 people), the *I*^*2*^ heterogeneity test showed a high heterogeneity (*I*^*2*^ = 99.03), and accordingly, random effects method was used to analyze the results. Considering the meta-analysis, the pooled global prevalence of isoniazid resistant TB was found to be 15.7% (95% *CI*: 13.7–17.9%). The study of publication bias in the studies through the Begg and Mazumdar correlation test showed the existence of publication bias in the studies (*P* = 0.02) (Table 1) (Figs. S3, S4 in Additional file 2).

### Rifampin resistant TB

In the review of 109 studies that had researched rifampin resistant TB (sample size of 215,660 people), the *I*^*2*^ heterogeneity test showed a high heterogeneity (*I*^*2*^ = 98.9), and similarly, random effects method was used to analyze the results. Based on the meta-analysis, the pooled global prevalence of rifampin- resistant TB was found as 9.4% (95% *CI*: 7.8–11.2%). The study of publication bias in the studies through the Begg and Mazumdar correlation test indicated the existence of publication bias in the studies (*P* = 0.00045) (Table 1) (Figs. S5, S6 in Additional file 2).

### Single drug resistant TB

In the review of 35 studies with a focus on single drug resistant TB (sample size of 45,147 people), the *I*^*2*^ heterogeneity test showed a high heterogeneity (*I*^*2*^ = 98.5). Hence, random effects method was used to analyze the results. Considering the meta-analysis results, the pooled global prevalence of single drug resistant TB was found as 11.8% (95% *CI*: 9.2–15.2%). The study of publication bias in the studies through the Begg and Mazumdar correlation test showed the absence of publication bias in the studies (*P* = 0.139) (Table 1) (Figs. S7, S8 in Additional file 2).

### Extensive drug resistant TB

In the review of 56 studies on extensive drug resistant TB (sample size of 350,420 people), the *I*^*2*^ heterogeneity test showed high heterogeneity (*I*^*2*^ = 98.8), and therefore, random effects method was used to analyze the results. Considering the meta-analysis results, the pooled global prevalence of extensive drug resistant TB was found to be 2.5% (95% *CI*: 2–3%). The study of publication bias using the Begg and Mazumdar correlation test indicated the absence of publication bias in the studies (*P* = 0.938) (Table 1) (Figs. S9, S10 in Additional file 2).

Information in Table 2 outlines the subgroup analysis of the types of tuberculosis resistance among patients by gender, and by TB type. Accordingly, male patients have a higher prevalence in multi-drug resistant TB, Isoniazid resistant TB and Rifampin-resistant TB, compared to female patients, with prevalence of 20% (95% *CI*: 11.9–31.8%), 17.5% (95% *CI*: 9.6–29.8%), and 12.7% (95% *CI*: 5.7–25.9%) respectively. Given that the articles did not report gender-segregated data for single drug-resistant TB and extensively drug-resistant TB, the authors could not include these results in the subgroup analysis.

**Table 2:** Subgroup analysis to investigate types of tuberculosis resistance in patients with gender segregation

Type	Sex	Number of articles	Sample size	*I*^*2*^	Begg and Mazumdar correlation test	Prevalence % (95% *CI*)
Multi-drug resistant TB	Male	24	7240	98.6	*P* = 0.413	20 (11.9–31.8)
	Female	22	3948	95.6	*P* = 0.611	13 (8.3–19.8)
Isoniazid resistant TB	Male	10	5312	98.9	*P* = 0.21	17.5 (9.6–29.8)
	Female	9	4046	95.7	*P* = 0.602	11.6 (7.3–18)
Rifampin-resistant TB	Male	11	7555	99.1	*P* = 0.876	12.7 (5.7–25.9)
	Female	10	9637	96.6	*P* = 0.591	8.8 (5.6–13.7)

TB: Tuberculosis, *CI*: Confidence interval

## Discussion

Tuberculosis is a very common infection with a bacterial agent called Mycobacterium [22, 45, 180–182]. MDR-TB is a strain of Tuberculosis (TB) that is resistant to at least two of the most important anti-tuberculosis drugs (INH and RIF) [180–185].

This systematic review and meta-analysis was conducted to identify and review existing research works that had examined prevalence of different types of TB. It was also aimed to obtain pooled prevalence of TB types globally. Accordingly, we did not find a specific study on the prevalence of drug-resistant tuberculosis at the global level, despite the fact that there are many articles that have reported the prevalence of this disease at country level, or at most in a continent.

Considering the reported results of an all included studies, the global pooled prevalence of different types of drug-resistant tuberculosis, namely MDR, Isoniazid (INH), Rifampcin (RIF), and XDR were calculated as 11.6%, 15.7%, 9.4%, and 2.5%, respectively.

Eastern European countries, the Russia and Central Asian countries, and parts of China have a high rate of MDR-TB infection [184, 186]. In the study by Kindu Alem Mola et al., the authors reported that the level of MDR-TB in East Africa is higher than other regions globally [187]. In this work, based on the relevant reports from the World Health Organization (WHO) in 2015, the prevalence of global MDR TB in new and previous TB cases were 3.5% and 20.5%, respectively, while countries in southern regions of Africa have greater rates [187, 188].

The main reasons for the emergence of MDR TB globally numerous [187], and they are mostly related to living conditions [189], lifestyle [190], previous medical history [111, 191], history of diabetes [192, 193] and Human Immunodeficiency Viruses (HIV) infection [194]. A study conducted in Ethiopia shows that HIV infection is one of the most important factors associated with MDR TB [187, 195]. In addition, HIV patients, due to the length of hospitalization in hospitals with poorer hygiene and infection control, are more exposed to MDR TB and hence the rate of infection is higher among these patients [187]. In another study by Al-Derraji et al. [187, 196], the incidence of MDR TB among HIV-positive patients was reported to be 20% higher compared to that of HIV-negatives [187].

In densely populated and poor families, the spread of TB disease is also more prevalent [187]. According to the literature, unhealthy or poor lifestyles which entail alcohol abuse, smoking, drug use, etc. are the main risk factors related to the spread of MDR TB [187]. It was also stated that smokers, especially men, are more likely to be infected with MDR TB compared to female smokers [187, 197–199].

According to an article by Jilani Talha et al., tuberculosis complications are usually seen more among elderly patients, young children, people with severe respiratory disorders or patients who do not receive proper treatment. Accordingly, patients who do not receive proper treatment are more exposed to tuberculosis complications. Some of these complications are acute respiratory distress syndrome, extensive lung destruction, empyema, pneumothorax, disseminated tuberculosis infection (including tuberculosis meningitis), bronchiectasis, fibrothorax, aspergilloma, and hemoptysis [200].

According to a study conducted by Jilani et al., with a focus on treating active tuberculosis, a combination of drugs is required during the two intensive and the continuous phases; the first-line drugs that are the most common regimen for tuberculosis treatment include: (1) isoniazid, (2) rifampin, (3) ethambutol, and (4) pyrazinamide [200].

The intensive phase in the treatment of Tuberculosis includes the combination of the above 4 drugs that are prescribed for 2 months, yet the continuation phase includes the combination of isoniazid and rifampin for an additional 4 months. The second line drugs include: (1) Injectable aminoglycoside: streptomycin, amikacin, kanamycin; (2) Injectable polypeptides: viomycin and capreomycin; (3) Fluoroquinolones: levofloxacin, gatifloxacin, ofloxacin and moxifloxacin, and (4) Para-amino salicylic acid, ethionamide, cycloserine, prothionamide, trazodone, linezolid [200].

In a study conducted, the side effects of each anti-tuberculosis drug were described as follows: (1) Isoniazid: liver damage (fatigue, nausea, lethargy, abdominal pain, and vomiting), skin rash, numbness, headache and tingling of limbs; (2) Rifampin: jaundice, arthralgia (joint stiffness), and fever; (3) Ethambutol: visual impairment including blurred or reduced vision and blindness, liver damage, headache, and nausea, and (4) Pyrazinamide: nausea, painful or swollen joints, and liver damage [200]. According to the reported results of the same study, the highest prevalence of multi-drug resistant tuberculosis was reported in males.

Considering the ratio of infections among males vs females, one study reported that the split between males and females with multi-drug resistant tuberculosis was 70.4% and 29.6% respectively [201], In a study conducted in patients with resistant tuberculosis in Ghana, the ratio of males and females was 69.6% and 30.4% respectively [202], whilst in another study conducted in Egypt, the ratio of males and females was reported as 67.5% and 32.5%, respectively [203]. Moreover, in a similar research work conducted in Ethiopia 65.3% male and 34.7% female has multi drug resistant TB [204].

Our study shows that different strains of Tuberculosis, including drug-resistant TBs, have a high prevalence. On the other hand, these strains can be treated, and there are similar strategies and interventions to control existing and new infections. Considering the complications that this disease may cause, its control and management are vital, since it would be possible to reduce the Tuberculosis induced mortality rate through controlling its different strains.

The main limitation of the present meta-analysis is related to the significant publication bias among the identified studies, and therefore, the results should be considered with caution. Moreover, it is recommended that future meta-analysis studies in this field are conducted using more keywords and databases to potentially eliminate this bias.

## Conclusions

According to the results of the present study, the global prevalence of multidrug-resistant, mono drug-resistant, isoniazid, and rifampicin tuberculosis are 11.6%, 11.8%, 15.7%, and 9.4%, respectively. The results of this study can offer some consistency to the heterogeneous results from studies conducted around the world and provide reliable insights to health policymakers. Such insights would be instrumental to devise appropriate preventive, therapeutic and diagnostic measures.

## Supplementary Information

**Additional file 1: Table S1.**Summary of Characteristics of Included Studies of Prevalence of MDR-TB.** TableS2.** Summary of Characteristics of Included Studies of Prevalence of Isoniazid Resistant-TB*.***Table S3.** Summary of Characteristics of Included Studies of Prevalence of Rifampcin Resistant-TB.**Table S4.**Summary of Characteristics of Included Studies of Prevalence of Single Drug Resistant-TB.**Table S5.**Summary of characteristics of included studies of prevalence of XDR-TB.**TableS6.** Summary of characteristics of included studies of prevalence of pre-XDR TB.

**Additional file 2: Figure S1.** Forest plot of the global prevalence ofmulti-drug resistant TB based on the random effects method. **Figure S2.** Funnel plotof publication bias in reviewed studies. **Figure S3.**Forest plot of global prevalence of isoniazid resistant TB based on randomeffects method. **Figure S4.** Funnel plot of publication biasin reviewed studies. **Figure S5.** Forest plot ofglobal prevalence of rifampin-resistant TB based on random effects method. **FigureS6.**Funnel plot of publication bias in reviewed studies. **FigureS7.** Forest plot of globalprevalence of single drug resistant TB based on random effects method. **FigureS8.**Funnel plot of publication bias in reviewed studies.**Figure S9.** Forest plot of global prevalence of extensively drugresistant TB based on random effects method. **Figure S10.** Funnel Plot of Publication Bias in ReviewedStudies.

## Data Availability

Datasets are available through the corresponding author upon reasonable request.

## Abbreviations

DST: Drug susceptibility testing
WGS: Whole genome sequencing
RFLP: Restriction fragment length polymorphism
ZN: Decontamination and Ziehl–Neelsen
CXR: Chest X-ray
AST: Antimicrobial susceptibility testing
*CI*: Confidence interval
TB: Tuberculosis
