# Supplementary material for: Global prevalence of drug-resistant tuberculosis: a systematic review and meta-analysis
Source: Infect Dis Poverty. 2023 May 25;12:57. doi: 10.1186/s40249-023-01107-x (PMC10210422; doi:10.1186/s40249-023-01107-x)
Supplement: Supplementary file 1 — Additional file 1: Table S1.Summary of Characteristics of Included Studies of Prevalence of MDR-TB. TableS2. Summary of Characteristics of Included Studies of Prevalence of Isoniazid Resistant-TB.Table S3. Summary of Characteristics of Included Studies of Prevalence of Rifampcin Resistant-TB.Table S4.Summary of Characteristics of Included Studies of Prevalence of Single Drug Resistant-TB.Table S5.Summary of characteristics of included studies of prevalence of XDR-TB.TableS6. Summary of characteristics of included studies of prevalence of pre-XDR TB. [file 40249_2023_1107_MOESM1_ESM.docx]

**Table S1:** Summary of Characteristics of Included Studies of Prevalence of MDR-TB.

| Author | Year | Region | Age | Time Horizon of the Methodology | Total sample size | Men sample size | Women sample size | Total Prevalence of multi-drug resistant TB | Prevalence of multi-drug resistant TB in men | Prevalence of multi-drug resistant TB in women | Instrument |
| --- | --- | --- | --- | --- | --- | --- | --- | --- | --- | --- | --- |
| Wang et al (11) | 2010 | shanghai | ≥65, <65 | cross sectional | 264 |  |  | 4.10% | - | - | - |
| Wang et al (11) | 2010 | Ningbo | ≥65, <65 | cross sectional | 254 |  |  | 11.60% | - | - | - |
| Timire et al (12) | 2019 | Zimbabwe | median age:34 | cross sectional | 1272 | 766 | 506 | 1.96% | - | - | survey questionnaire eliciting socio-demographic |
| Ahmad et al (13) | 2015 | Pakistan | 30.4 ± 14.4 ,10->60 | cross sectional | 289 | 108 | 135 | 2.07% | - | - | - |
| Ofonakara et al (14) | 2019 | Ebonyi state | 31–40 | cross sectional | 351 | 6 | 2 | 2.30% | 75% | 25% | Spoligotyping, DNA isolation and sequencing of loci |
| Hu et al (15) | 2015 | Rural China | 53 ± 15.1 | cross sectional | 1332 | 102 | 48 | 11.26% | 7.65% | 3.60% | Spoligotyping, DNA isolation and sequencing of loci |
| Mazahir et al (16) | 2017 | India | 15–52 | cross sectional | 80 | 12 | 9 | 26.25% | 15% | 11.25% | Detailed demographic, socio-economic and treatment history |
| Ayaz et al (17) | 2012 | Karachi, Pakistan | >27,34(±13) | cross sectional | 1004 | 18 | 25 | 4.30% | 1.79% | 2.49% | - |
| Lapphra et al (18) | 2012 | Bangkok,Thailand |  | A retrospective study2008-2011 | 230 |  |  | 5.70% |  |  | - |
| Yang et al (19) | 2014 | Northeast China | 15–44 | cross sectional | 205 | 12 | 2 | 6.80% | - | - | basis of clinical examination, CXR, sputum smear microscopy and sputum culture |
| Magula et al (20) | 2014 | sub-Saharan Africa | - | cross sectional | 163 | - | - | 9.50% | - | - |  |
| Mahabeer et al (21) | 2016 | KwaZulu-Natal, South Africa | less than 5 years old | A retrospective descriptive analysis2012-2014 | 903 | - | - | 11-16% | - | - | automated Mycobacterial Growth Indicator Tube 960 system |
| Singhal et al (23) | 2017 | Punjab state of India | median age:37 | cross sectional | 557 | - | - | 16.90% | - | - | immunochromatographic test |
| Prakash et al (24) | 2015 | Sahariya tribe of North central India | 38.26 ± 13.67 | cross sectional | 185 | 140 | 45 | 11.35% | 10.71% | 13.30% | drug sensitivity tests, field-based investigations |
| Prakash et al (24) | 2015 | non-tribe of North central India | 39.74 ± 15.49 | cross sectional | 89 | 72 | 17 | 6.74% | 6.94% | 5.88% | drug sensitivity tests, field-based investigations |
| Dujaili et al (25) | 2013 | Baghdad, Iraq | - | cross sectional | 2968 | 1810 | 1158 | 3.47% | - | - | Databases of Thoracic and Respiratory Diseases |
| Santos et al (26) | 2010 | Midwestern BRAZIL | 1 to 77 | cross sectional | 132 | 106 | 26 | 6.10% | - | - |  |
| Kontsevaya et al (27) | 2016 | Eastern Europe | - | cross sectional | 512 | - | - | 16.40% | - | - | chi-square test with p b 0.05 |
| Bhembe et al (28) | 2020 | Eastern Cape, South Africa | 0-14, 15-29, 30-44, 45-59, 60 and above | cross sectional | 3810 | - | - | 4.80% | - | - | antibiotic susceptibility test |
| Ghebremichael et al (29) | 2008 | Sweden | - | cross sectional | 400 | 199 | 201 | 11.50% | - | - | spoligotyping, molecular fingerprinting, restriction fragment length polymorphism (RFLP) |
| Montoro et al (30) | 2006 | Cuba | - | cross sectional | 1528 | - | - | 0.07% | - | - | Drug susceptibility testing (DST^[[1]](#footnote-1)^) |
| Pardini et al (31) | 2009 | Abkhazia (Georgia) | Median age:41 | cross sectional | 326 | 252 | 74 | 21% | - | - | Drug susceptibility testing (DST), molecular typing |
| Jiao et al (32) | 2015 | China | average age:6.8 | cross sectional | 100 | 57 | 43 | 22% | - | - | Drug susceptibility testing |
| Jiao et al (32) | 2015 | China | average age:16.9 | cross sectional | 159 | 83 | 76 | 22.01% | - | - | Drug susceptibility testing |
| Jiao et al (32) | 2015 | China | average age:40.8 | cross sectional | 191 | 122 | 69 | 30.89% | - | - | Drug susceptibility testing |
| Brandao et al (33) | 2020 | Sao Paulo, Brazil | 37 ± 13 y | cross sectional | 283 | 206 | 77 | 30.03% | - | - | Xpert tests, Phenotypic drug susceptibility testing, Gene sequencing,2.5. Molecular typing |
| Van Rie et al (34) | 2000 | Cape Town, South Africa |  | cross sectional | 63 |  |  | 71.42% | - | - | RFLP |
| Chand et al (35) | 2000 | Pune, India | Median age:36 years | cross sectional | 1120 | 130 | 62 | 0.71% | - | - | Ziehl Neelsen method |
| Ismail Et al (36) | 2018 | South Africa | ≥18 years old | cross sectional | 101422 |  |  | 2.80% | - | - | population proportionate, cluster sampling design |
| Daniel Et al (37) | 2011 | Southwest, Nigeria | Men:35.9±10.0, Women:33.5±9.3 | retrospective study | 88 | 50 | 38 | 76.40% | 50.00% | 44.73% | Drug susceptibility test |
| Wang et al (38) | 2002 | Henan, China | - | cross sectional | 1372 | 973 | 399 | 23.32% | 22.09% | 42.85% | Standard drug susceptibility testing |
| Hannan Et al (39) | 2001 | Lisbon | 20–29 ,30–39, +40 | A retrospective epidemiological and laboratory study | 134 | - | - | 70.80% | - | - | DNA fingerprinting, Susceptibility testing |
| Wu et al (40) | 2017 | Zhejiang Province, China | < 25 ,25–45,45–65, ≥ 65 | cross sectional | 936 | 656 | 280 | 5.02% | 72.30% | 27.70% | questionnaires, drug susceptibility testing (DST) |
| Phyu et al (41) | 2005 | Yangon, Myanmar | new cases: 36.69 ± 13.2 previously treated: 39.3 ± 12.6 | cross sectional | 357 | 246 | 111 | 8.12% | - | - | Drug susceptibility testing |
| Hu et al (42) | 2008 | Rural China, Deqing | 56 ± 21 | cross sectional | 182 | 11 |  | 7.14% | 6.04% | - | Questionnaire interviews, Bacteriological test |
| Hu et al (42) | 2008 | Rural China, Guanyun | 58 ± 17 | cross sectional | 217 | 40 |  | 20.70% | 20.70% | - | Questionnaire interviews, Bacteriological test |
| Shah et al (43) | 2018 | Mumbai, India | 3 months to <16 years | retrospective cross-sectional study | 196 | - | - | 34.18% | - | - | GeneXpert, PA^[[2]](#footnote-2)^,drug susceptibility testing (DST) |
| Ignatova Et al (44) | 2006 | Tula Region, Russia | 20–56, Mean age:35.9 | cross sectional | 87 | 87 |  | 77.00% | 77.00% |  | drug susceptibility testing |
| Kamolwat et al (46) | 2021 | Thailand | 45–54 | cross sectional | 1501 |  |  | 1.39% | - | - | Microscopy examination, mycobacterial culturists |
| Yi Hu et al (47) | 2013 | Rural China | - | cross sectional | 380 | 267 | 113 | 11.11% | - | - | Species identification and drug susceptibility testing |
| Huo et al (48) | 2019 | China | - | cross sectional | 542 | 340 | 202 | 27.67% | - | - |  |
| Zhao et al (49) | 2014 | Hunan, China | - | cross sectional | 171 | 33 | 10 | 25.14% | - | - | drug susceptibility testing (DST), Spoligotyping |
| Daum et al (50) | 2018 | Ukraine | Mean Age:42y | cross sectional | 75 | 63 | 12 | 53.30% | - | - | DST |
| Dinic et al (51) | 2012 | Nigeria | - | cross sectional | 224 | 130 | 94 | 3.57% | - | - | Multiplex-nested PCR ^[[3]](#footnote-3)^and DNA sequencing of resistance genes |
| Agarwal et al (52) | 2010 | Malatya, Turkey | 1–89, Mean Age:33.9 y, Median of 32 years | cross sectional | 397 | 228 | 159 | 4.53% | - | - | drug susceptibility testing |
| Djuretic et al (53) | 2015 | United Kingdom | 15–44 years | cross sectional | 25217 | 14214 | 10049 | 1.20% | - | - | - |
| Xin-Tong et al (54) | 2017 | Dalian, China | 0–30 ,31-59, ≥ 60 | cross sectional | 3522 in totalin total (2261new & 1291 previously treated) | 2680 | 872 | 10.2% in totalin total (5.8 % new &17.7% previously treated) | - | - | DST |
| Fairlie et al (55) | 2011 | Johannesburg, South Africa | Median Age:25 | cross sectional | 148 |  |  | 8.80% | - | - | Drug susceptibility testing (DST |
| Sharaf et al (56) | 2011 | Sudan | < = 30 ,31-44,> = 45 | cross sectional | 235 | 175 | 60 | 4.90% | 12% | 8.30% | Spoligotyping, Drug susceptibility testing |
| Mekonnen et al (57) | 2015 | Metema and west Armachiho, Northwest Ethiopia | median:29y | cross sectional | 124 | 80 | 44 | in totalin total 5.7% (2.3%new & 13.9% previously treated) | 7.50% | 2.27% | Socio-demographic and possible risk factor data, drug susceptibility testing |
| Diallo et al (58) | 2017 | Chad | 12–70years | cross sectional | 311 | 224 | 87 | (0.9% new & 3.5% previously treated) | - | - | Drug-susceptibility testing (DST) |
| Juma et al (59) | 2019 | Tanzania | 33 ± 10years | cross sectional | 91 | 23 | 68 | 32.90% | - | - | Sputa processing and PZA ^[[4]](#footnote-4)^susceptibility testing |
| Ogari et al (60) | 2019 | Nairobi, Kenya | mean age: 26.4 ± 19.4 y | cross sectional | 132 | 72 | 60 | 0.80% | - | - | Decontamination and Ziehl-Neelsen (ZN) sputum smear microscopy |
| Saldanha et al (61) | 2019 | Western India | Median age:41.5y | cross sectional | 200 | 134 | 66 | in totalin total12.5% (8.8% new & 23.1% previously treated) | - | - |  |
| Huo et al 2005(48) | 2019 | China | - | cross sectional | 273 | 45 | 228 | 24.20% | - | - | Conventional drug susceptibility testing and Mycobacterium species identification |
| Huo et al 2015(48) | 2019 | China | - | cross sectional | 269 | 69 | 200 | 31.20% | - | - | Conventional drug susceptibility testing and Mycobacterium species identification |
| Gehre et al (62) | 2016 | West Africa | - | cross sectional | 974 |  |  | (6%new & 35% previously treated) | - | - | First- and second-line drug susceptibility testing |
| Diriba et al (63) | 2019 | Ethiopia | (97.9% age>15 years /2.1% age <15 years) | cross sectional | 329 | 243 | 86 | in totalin total 11.6% (4.3% new & 6.7% previously treated) | - | - | - |
| Cox et al new (64) | 2010 | Khayelitsha, South Africa | - | cross sectional | 271 | 155 | 116 | 3% | - | - | Drug susceptibility testing (DST) |
| Cox et al previously treated (64) | 2010 | Khayelitsha, South Africa | - | cross sectional | 264 | 167 | 96 | 6.90% | - | - | Drug susceptibility testing (DST) |
| Hom et al (65) | 2012 | Durban, South Africa | median age:36 | cross sectional cohort study | 191 | 92 | 99 | 5.10% | - | - |  |
| Porwal et al (66) | 2013 | Delhi Region | 31.7 ± 11.27 | cross sectional | 611 | 260 | 152 | 79.05% | - | - | DST |
| Isaakidis et al (67) | 2014 | Metropolitan Mumbai, India | median age:35.0 | cross sectional | 1724 | 1042 | 671 | 38% | - | - |  |
| Ullah et al (68) | 2016 | Pakistan | mean age: 39.7± 18.5 years | cross sectional | 2367 | 1238 | 1129 | 9.30% | 9.20% | 9.50% | Drug susceptibility testing |
| Mesfin et al (69) | 2018 | Addis Ababa, Ethiopia | average age:34.4y | cross sectional | 226 | 133 | 93 | 39.40% | 41.60% | 58.40% | - |
| Kigozi et al (70) | 2018 | Uganda | - | cross sectional | 97 |  |  | 39.17% | - | - | - |
| Shibabaw et al (71) | 2020 | Amhara region, Ethiopia | median age: 30 y | cross sectional | 211 | 133 | 78 | 87% | - | - | GeneXpert MTB^[[5]](#footnote-5)^/RIF ^[[6]](#footnote-6)^assay, genotypic and phenotypic drug susceptibility testing (DST). |
| Gilad et al (72) | 2000 | Southern Israel | mean age:52 ± 20 y | cross sectional | 249 | 138 | 111 | 8.50% | - | - | - |
| Ramzan et al (73) | 2004 | Asia | - | cross sectional | 47 | - | - | 2.13% | - | - | - |
| Um SJ et al (74) | 2011 | Korea | - | cross sectional | 373 | - | - | 10.40% | - | - | - |
| Diandé et al (75) | 2019 | Burkina Faso | 2-39, ≥40 | cross sectional | 1141 | 845 | 295 | 83% | - | - | Drug susceptibility testing |
| Becerril-Montes et al (76) | 2013 | Mexico/United States | 15–65 | - | 139 | 82 | 57 | 17.27% | - | - | Drug susceptibility testing (DST) for first-line anti-TB drugs |
| Bastos et al (77) | 2012 | Rio de Janeiro, Brazil | ≥18 years old | cross sectional | 209 | 131 | 78 | 0.50% | - | - | Drug susceptibility testing |
| Micheletti et al (78) | 2014 | Porto Alegre, Brazil | mean age:36 years | cross sectional | 299 | 221 | 78 | 4.70% | - | - | drug susceptibility testing |
| Zhao et al (79) | 2019 | China | mean age: 40.4 years | a retrospective cross-sectional study | 189 | 132 | 57 | 9.50% | - | - | DST |
| Migliori et al (80) | 2007 | Italy & Germany | - | cross sectional | 2888 in totalin total (2140 Italy & 748 Germany) | - | - | 4.4%in totalin total (3.88% Italy & 5.75% Germany) | - | - | - |
| Deng et al (81) | 2011 | China | mean age:40.1 ± 18.9 year | a retrospective cross-sectional study | 989 | 648 | 341 | 18.70% | - | - | DST |
| Wallengren et al (82) | 2011 | KwaZulu-Natal, South Africa | - | a retrospective cross-sectional study | 119218 | - | - | 2.30% | - | - | drug-sensitivity testing |
| Wang et al (1999) (65) | 2012 | Zhejiang Province, China | 0–14, 15-64, >65 | cross sectional | 1013 | 723 | 290 | 8.60% | 8.30% | 9.30% | Drug sensitivity tests |
| Wang et al (2004) (65) | 2012 | Zhejiang Province, China | 0–14, 15-64, >65 | cross sectional | 984 | 730 | 254 | 7.60% | 8.40% | 5.50% | Drug sensitivity tests |
| Wang et al (2008) (65) | 2012 | Zhejiang Province, China | 0–14, 15-64, >65 | cross sectional | 938 | 646 | 292 | 6.00% | 6.20% | 5.50% | Drug sensitivity tests |
| El Achkar et al (83) | 2019 | Lebanon, | 34 ± 14 | cross sectional | 250 | 128 | 122 | 1.20% | - | - | Xpert testing |
| Lee et al (84) | 2009 | Korea | median age:21 yr | retrospective | 148 | 148 | 0 | 8.10% | - | - | drug susceptibility test |
| Buyankhishig et al (85) | 2011 | Mongolia |  | cross sectional | 850 | 507 | 343 | 7.50% | 68.70% | 31.30% | - |
| Seddon et al (86) | 2012 | Cape Town, South Africa | a median age of 26 months | cross sectional | 292 | 156 | 136 | 8.90% | - | - | DST |
| Bojorquez et al(87) | 2012 | Mexico | (42.9 ± 47.7) | cross sectional | 2121 | 64.50% | 35.50% | 2.80% | - | - | DST |
| Pwei et al (88) | 2017 | Myanmar | - | a cross-sectional, retrospective study | 94 |  |  | 94.68% | - | - | DST |
| Smith et al (89) | 2017 | Hlabisa subdistrict, KwaZulu-Natal | 25–34 years | cross sectional | 489 | 239 | 250 | 97.80% | - | - | Xpertw MTB/FIR testing |
| Alikhanova et al (90) | 2014 | Republic of Azerbaijan | - | cross sectional | 789 | - | - | 17.49% | - | - | A standard questionnaire |
| Tasbiti et al (91) | 2017 | Tehran, Iran | 46.6 ± 18.8 | a retrospective cross-sectional study | 1442 | - | - | 2.30% | - | - | Sputum culture and drug susceptibility testing (DST) |
| Cox et al (92) | 2004 | Central Asia | median age :31 y | a cross-sectional survey | 213 | 60% | 40% | 26.80% | - | - |  |
| Otokunefor et al (93) | 2018 | Port Harcourt, Nigeria | - | cross sectional | 609 | 399 | 210 | 3.30% | - | - | DST |
| Roshdi Maleki et al (94) | 2012 | East Azerbaijan province of Iran | - | cross sectional | 230 | 230 | 0 | 3.48% | - | - | drug susceptibility test |
| Israel et al (95) | 2018 | Zanmi Lasante Network | median age: 32 | cross sectional | 222 | 53.15% | 46.84% | 9.01% | - | - | Gene xpert testing |
| Wang et al (96) | 2016 | China |  |  | 2794 | 1937 | 857 | 5.70% | - | - | DST |
| Wang et al (97) | 2010 | China |  |  | 967 | 642 | 325 | 19.44% | - | - |  |
| Laghari et al (98) | 2019 | Pakistan | mean age was 12.84 ± 2.54 years | cross sectional | 50 | 32 | 18 | 36% | - | - |  |
| Afroz et al (99) | 2014 | Narshingdi, Bangladesh | range: 25–34 years | cross sectional | 152 | 65 | 87 | 11.18% | - | - |  |
| Faridi et al (100) | 2018 | Aligarh Region | - | - | 514 | - | - | 9.14% | - | - |  |
| F. Aguiar et al (101) | 2008 | Rio de Janeiro, Brazil | - | retrospective cross-sectional study | 350 | 223 | 127 | 4.30% | - | - | drug susceptibility tests |
| Rashedi et al (102) | 2015 | Republic of Azerbaijan | - | cross sectional | 32 | - | - | 62.50% | - | - | drug susceptibility tests |
| Rashedi et al (102) | 2015 | North-West of Iran | - | cross sectional | 48 | - | - | 2.10% | - | - | drug susceptibility tests |
| Akhtar et al (103) | 2016 | Punjab, Pakistan | The mean age was 32 (±13.5 SD) years. | cross sectional | 1250 | 664 | 586 | 69% | - | - | DST |
| Anunnatsiri et al (104) | 2005 | Thailand | 47.2 ± 17.7 years | retrospective survey | 226 | 150 | 76 | 2.80% | - | - | - |
| Surucuoglu et al (105) | 2005 | Western Turkey | The median age of the patients was 45 ± 15.24 years for males and 44 ± 18.79 years for females. | cross sectional | 355 | 273 | 82 | 7.30% | 9% | 1% | DST |
| Bhat et al (106) | 2014 | Saharia tribe of central India | - | cross sectional | 475 | - | - | 4% | - | - |  |
| Kinjal et al (107) | 2019 | Jamnagar District | - | cross sectional | 1332 | - | - | 5.57% | - | - | DST, LPA |
| Jacobs et al (108) | 2019 | Brazil | 87.0% were between 20 and 59 years old | descriptive study | 1574 | 1111 | 463 | 50.90% | - | - | - |
| Dodd et al (109) | 2016 | - | - | mathematical model | 58000 | - | - | 2.90% | - | - | - |
| Abouyannis et al (110) | 2014 | Malawi | mean ages of 40.7 and 36.4 years | cross sectional | 1777 in total (1196 new & 581 retreatment) | 1777 | 0 | 1.85% in total (0.4 % new & 4.8% retreatment) | - | - | - |
| Abdella et al (111) | 2015 | Jimma, Southwest Ethiopia | the mean age of 31.67 ± 10.02 y | cross sectional study | 79 | 48 | 31 | 31.40% | - | - | DST, socio demographic |
| Weyer et al ( 112) | 2007 | Africa | 0-64, +64 | cross-sectional | 5866 | 3746 | 2115 | 2.90% | - | - | - |
| Soliman et al (113) | 2021 | - | - | cross sectional | 1069 |  |  | 7.16% | - | - | DST, gene xpert |
| Villa-Rosas et al (114) | 2015 | Mexico | Mean age was 38.5 ± 13.9 | cross sectional | 214 | 146 | 68 | 3.90% | - | - | DST |
| Aia et al (115) | 2016 | Papua New Guinea | median age: 28 y (males median age :30y & females median age: 27 y) | cross sectional | 1182 | 590 | 592 | 2.70% | - | - | - |
| Cox et al (64) | 2010 | Khayelitsha, South Africa | (new cases: 32 years & previously treated: 36 years) | A cross-sectional survey | 535 | 322 | 212 | (3.3% new & 7.7% previously treated) | - | - | DST |
| Hang et al (116) | 2013 | Hanoi, Viet Nam | median age: 38.6 years | - | 489 | 386 | 103 | 4.50% | - | - | DST, spoligotyping |
| Minion et al (117) | 2013 | Canada | - | cross sectional | 15993 | 8784 | 7209 | 1.10% | - | - | DST |
| Shibabaw et al (71) | 2020 | Amhara region, Ethiopia | The median age of the participants was 30 years | cross sectional study | 211 | 133 | 78 | 87% | - | - | genotypic and phenotypic drug susceptibility testing (DST), GeneXpert MTB/RIF assay |
| Gallo et al (118) | 2017 | Sao Paulo, Brazil | mean age :39.5 ± 13.6 years | cross sectional | 313 | 162 | 59 | 70.60% | 73.30% | 26.70% | DST |
| Bedaso et al (119) | 2021 | Bale Zone, Oromia Region, Ethiopia | - | A descriptive study | 43 | 14 | 29 | 30.20% | - | - | DST |
| Anwaierjiang et al (120) | 2021 | Southern Xinjiang, China | mean age of 50.4 (±19.3) years | cross sectional | 346 | 167 | 179 | 14.74% | - | - | phenotypic drug susceptibility testing, whole genome sequencing (WGS^[[7]](#footnote-7)^) |
| Patil et al(121) | 2019 | Western Maharashtra, India | - | prospective study | 1154 | - | - | 5.55% | - | - | GeneXpert MTB/RIF assay |
| Kulkarni et al (122) | 2020 | Maharashtra | 15–55 years | retrospective study | 25191 | - | - | 1.26% | - | - | - |
| Adwani et al (123) | 2016 | Mumbai, India | - |  | 227 | 113 | 114 | 39.20% | 34.50% | 43.86% | DST |
| Dagne et al (124) | 2021 | Ethiopia | most frequent age-group was 25–34 years | A health facility-based cross-sectional study | 209 | 140 | 64 | 61.90% | - | - | Susceptibility testing |
| Micheni et al (125) | 2021 | southwestern Uganda | The median age of all patients was 36 years | cross sectional study | 283 | 207 | 76 | 8.50% | - | - | smear microscopy or Cepheid GeneXpert |
| Anees et al (126) | 2019 | Peshawar,Khyber PakhtonKhwa province of Pakistan | Mean age of all patients were 29.80 ± 16.32 | cross sectional | 102 | - | - | 34.31% | 23.85% | 25.86% | gene expert procedure |
| Lai et al (127) | 2008 | Taiwan, China | - | retrospective analysis | 2625 | - | - | 5.70% | - | - |  |
| Lukoye et al (128) | 2013 | Uganda | median age :34.6 years | cross sectional | 1537 in total (1397 new & 140 previously treated) | 1018 | 519 | (1.4% new & 12.1 % previously treated) | - | - | Culture and drug susceptibility testing |
| Oyedeji et al (129) | 2020 | Osun-State, Nigeria | ≤15, 16-55, ≥56 | cross sectional | 207 | 103 | 104 | 2.90% | 2.91% | 2.88% | GeneXpert test, Drug susceptibility testing |
| Masood et al (130) | 2019 | Karachi, Pakistan | - | A retrospective study | 315 | - | - | 64.10% | - | - | Chi-square test |
| Safwat et al (131) | 2011 | Abbassia Chest Hospital, Egypt | mean age: 36.8years | retrospective study | 180 | 130 | 50 | (0.31% at 2006, 0.38% at 2007, 0.64% at 2008, 0.48% at 2009) | - | - | DST |
| Abebe et al (132) | 2012 | southwestern Ethiopia | median age: 26.0 (± 10.7) years | across sectional study | 136 | 78 | 58 | 1.50% | - | - | DST |
| Yoshiyama et al (133) | 2001 | northern Thailand |  | cross sectional | 1077 | 748 | 329 | 6.30% | - | - | DST, Ziehl-Neelsen method |
| Dewan et al (134) | 2005 | Republic of Lithuania | The median age was 45years | - | 1163 in total (818 new & 345 previously treated) | 854 | 309 | (9.3% new & 54.2% previously treated) | - | - |  |
| Pleumpanupat et al(135) | 2003 | Thailand | mean age: 32.81 ± 7.69 years | cross sectional | 154 |  |  | 19.50% | - | - | DST |
| Sidze et al (136) | 2014 | Central Region of Cameroon | mean age :33.9 ± 12.3 years | cross sectional | 665 | 390 | 275 | 1.10% | - | - | DST |
| Mekonnen et al (137) | 2014 | Amhara National Regional State, Ethiopia | mean age: 35.6 years | cross sectional | 606 | 363 | 243 | 15.30% | - | - | - |
| Castiñeiras et al (138) | 2006 | Castilla-León, Spain | - | cross sectional | 985 | - | - | 0.10% | - | - | DST |
| Elmi et al (139) | 2014 | Malaysia | mean age: 41.78(17.13) | cross sectional | 139 | 23 | 46 | 49.60% | - | - |  |
| Amala (140) | 2019 | Bayelsa State, Nigeria | age 20-39 had the highest prevalence of MDR TB | - | 429 | 208 | 221 | 5.80% | 3.30% | 2.60% | GenXpert |
| Brito et al (141) | 2010 | Rio de Janeiro, Brazil | <40, 40-60,  >60 | cross sectional | 595 | 409 | 186 | 7.40% | - | - | DST |
| Wang et al (142) | 2021 | China | - | - | 391 | - | - | 68.80% | - | - | DST |
| Xiang et al (143) | 2011 | Chongqing, China | mean age: 42.3 years | cross sectional | 1089 | 728 | 361 | 7.30% | - | - | DST |
| Yang et al (144) | 2015 | China | - | - | 1771 in total (1174 new & 597 retreated) | - | - | (8.6% new & 23.2% retreated) | - | - |  |
| Beltran et al (145) | 2011 | Sinaloa, Mexico | - | cross sectional | 671 | - | - | (1997-2002: 17.9%, 2003-2004 :18.2%) | - | - | Susceptibility test |
| Pang et al (146) | 2017 | China | median age: 42 years | A retrospective study | 9544 | 16 | 13 | 35.40% | - | - | DST |

**Table S2:** Summary of Characteristics of Included Studies of Prevalence of Isoniazid Resistant-TB

| Author | Year | Region | Age | Time horizon of the methodology | Total sample size | Men sample size | Women sample size | Total Prevalence of Isoniazid resistant TB | Prevalence of Isoniazid resistant TB in men | Prevalence of Isoniazid resistant TB in women | Instrument |
| --- | --- | --- | --- | --- | --- | --- | --- | --- | --- | --- | --- |
| Ayaz et al (17) | 2012 | Karachi, Pakistan | >27,32 (±16) | cross sectional | 1004 | 20 | 15 | 3.50% | 1.99% | 1.49% | - |
| Lapphra et al (147) | 2012 | Bangkok, Thailand | - | A retrospective study2008-2011 | 230 | - | - | 11.30% | - | - | - |
| Yang et al (19) | 2014 | Northeast China | 15-44 | cross sectional | 205 | - | - | 3.40% | - | - | basis of clinical examination, CXR, sputum smear microscopy and sputum culture |
| Mahabeer et al (21) | 2016 | KwaZulu-Natal, South Africa | less than 5 years old | A retrospective descriptive analysis2012-2014 | 903 | - | - | 3.40% | - | - | automated Maycobacterial Growth Indicator Tube 960 system |
| Singhal et al (23) | 2017 | Punjab state of India | median age:37 | cross sectional | 557 | - | -- | 7.10% | - |  | immunochromatographic test |
| Santos et al(26) | 2010 | Midwestern Brazil | 1 to 77 | cross sectional | 132 | 106 | 26 | 6.81% | -- |  | - |
| Ghebremichael et al (29) | 2008 | Sweden | - | cross sectional | 400 | 199 | 201 | 75% | -- |  | spoligotyping, molecular fingerprintin,restriction fragment length polymorphism(RFLP) |
| Pardini et al (31) | 2009 | Abkhazia (Georgia) | Median age:41 | cross sectional | 326 | 252 | 74 | 8.40% | - | - | Drug susceptibility testing (DST), molecular typing |
| Jiao et al (32) | 2015 | China | average age:6.8 | cross sectional | 100 | 57 | 43 | 29% | - | - | Drug susceptibility testing |
| Jiao et al (32) | 2015 | China | average age:16.9 | cross sectional | 159 | 83 | 76 | 30.18% | - | - | Drug susceptibility testing |
| Jiao et al (32) | 2015 | China | average age:40.8 | cross sectional | 191 | 122 | 69 | 44.50% | - | - | Drug susceptibility testing |
| Chand et al (148) | 2000 | Pune, India | median age:36 years | cross sectional | 1120 | 130 | 62 | 7.29% | - | - | Ziehl Neelsen method |
| Ismail Et al (149) | 2018 | South Africa | ≥18 years old | cross sectional | 101422 |  |  | 9.30% | - | - | population proportionate, clustersampling design |
| Wang et al (38) | 2002 | Henan, China | - | cross sectional | 1372 | 973 | 399 | 37.09% | - | - | Standard drug susceptibility testing |
| Phyu et al (41) | 2005 | Yangon, Myanmar | new cases: 36.69 ± 13.2 previously treated: 39.3 ± 12.6 | cross sectional | 357 | 246 | 111 | 27.17% | - | - | Drug susceptibility testing |
| Liu et al (150) | 1998 | New Jersey | <25 ,25–44 ,45-64,>65 | cross sectional | 1926 | 1207 | 719 | 10.60% | 11.10% | 9.90% | drug susceptibility testing, completion of chemotherapy, initial drug regimen, directly observed therapy |
| Shah et al (43) | 2018 | Mumbai, India | 3 months to <16 years | retrospective cross-sectional study | 196 | - | - | 72.90% | - | - | GeneXpert,LPA,drug susceptibility testing(DST) |
| Ignatova Et al (44) | 2006 | Tula Region, Russia | Mean Age :35.9 | cross sectional | 87 | 87 |  | 71.20% | 71.20% | - | drug susceptibility testing |
| Kamolwat et al (46) | 2021 | Thailand | 45–54 | cross sectional | 1501 | - | - | 10.39% | - | - | Microscopy examination, mycobacterial culture, DST |
| Yi hu et al (47) | 2013 | Rural China | - | cross sectional | 380 | 267 | 113 | 3.40% | - | - | Species identification and drug susceptibility testing |
| Huo et al (151) | 2018 | China | - | cross sectional | 542 | 340 | 202 | 34.50% | - | - | - |
| Zhao et al (49) | 2014 | Hunan, China | - | cross sectional | 171 | 33 | 10 | 35.67% | - | - | drug susceptibility testing (DST), Spoligotyping |
| Bruchfeld et al (152) | 2002 | Addis Ababa, Ethiopia | Mean Age :(clustered:29.3 non clustered:31.1) | cross sectional | 121 | 78 | 43 | 8.26% | - | - | Drug susceptibility testing |
| Dinic et al (51) | 2012 | Nigeria | - | cross sectional | 224 | 130 | 94 | 5.35% | - | - | Multiplex-nested PCR and DNA sequencing of resistance genes |
| Agarwal et al (153) | 2010 | Malatya, Turkey | Mean Age:33.9 y | cross sectional | 397 | 228 | 159 | 15.60% | - | - | drug susceptibility testing |
| Djuretic et al (53) | 2015 | United Kingdom | 15–44 years | cross sectional | 25217 | 14214 | 10049 | 5.70% | - | - | - |
| Fairlie et al (55) | 2011 | Johannesburg, South Africa | Median Age:60 | cross sectional | 148 | - | - | 14.20% | - | - | Drug susceptibility testing (DST |
| Sharaf Eldin et al (56) | 2011 | Sudan | < = 30 ,31-44,> = 45 | cross sectional | 235 | 175 | 60 | 10.40% | - | - | Spoligotyping, Drug susceptibility testing |
| Diallo et al (154) | 2017 | Chad | 12-70 years | cross sectional | 311 | 224 | 87 | (4.5% new & 9% previously treated) | - | - | Drug-susceptibility testing (DST) |
| Ogari et al (60) | 2019 | Nairobi, Kenya | mean age: 26.4 ± 19.4 y | cross sectional | 132 | 72 | 60 | 0.80% | - | - | Decontamination and Ziehl-Neelsen (ZN) sputum smear microscopy |
| Saldanha N et al (61) | 2019 | Western India | Median age:41.5y | cross sectional | 200 | 134 | 66 | 9% | - | - |  |
| Huo et al 2005 (151) | 2019 | China | - | cross sectional | 273 | 45 | 228 | 30.00% | - | - | Conventional drug susceptibility testing and Mycobacterium species identification |
| Huo et al 2015 (151) | 2019 | China | - | cross sectional | 269 | 69 | 200 | 39.00% | - | - | Conventional drug susceptibility testing and Mycobacterium species identification |
| Diriba et al (63) | 2019 | Ethiopia | (97.9% age>15 years /2.1% age <15 years) | cross sectional | 329 | 243 | 86 | 5.80% | - | - | - |
| Cox et al new (64) | 2010 | Khayelitsha, South Africa | - | cross sectional | 271 | 155 | 116 | 7.10% | - | - | Drug susceptibility testing (DST) |
| Cox et al previously treated (64) | 2010 | Khayelitsha, South Africa | - | cross sectional | 264 | 167 | 96 | 9.70% | - | - | Drug susceptibility testing (DST) |
| Ullah et al (155) | 2016 | Pakistan | mean age: 39.7± 18.5 years | cross sectional | 2367 | 1238 | 1129 | 9.90% | 9.70% | 9.90% | Drug susceptibility testing |
| Ombura et al (156) | 2016 | Mombasa, Kenya | median age:30 | cross sectional | 258 | 174 | 84 | 3.10% | 1.90% | 1.20% | Demographic and clinical data collection |
| Mesfin et al (69) | 2018 | Addis Ababa, Ethiopia | average age:34.4y | cross sectional | 226 | 133 | 93 | 48.70% | - | - | - |
| Kigozi et al (70) | 2018 | Uganda |  | cross sectional | 97 |  |  | 12.37% | - | - | - |
| Shibabaw et al (71) | 2020 | Amhara region, Ethiopia | median age: 30 y | cross sectional | 211 | 133 | 78 | 88.50% | - | - | GeneXpert MTB/RIF assay, genotypic and phenotypic drug susceptibility testing (DST). |
| Gilad et al (72) | 2000 | Southern Israel | mean age:52 ± 20 y | cross sectional | 249 | 138 | 111 | 16% | - | - | - |
| Ramzan et al (73) | 2004 | Asia | - | cross sectional | 47 | - | - | 20.50% | - | - | - |
| Um SJ et al(74) | 2011 | Korea | - | cross sectional | 373 | - | - | 18.20% | - | - | - |
| Becerril-Montes, P et al (157) | 2013 | Mexico/United States | 15–65years | - | 139 | 82 | 57 | 37.41% | - | - | Drug susceptibility testing (DST) for first-line anti-TB drugs |
| Bastos et al (158) | 2012 | Rio de Janeiro, Brazil | ≥18 years old | cross sectional | 209 | 131 | 78 | 9.60% | - | - | Drug susceptibility testing |
| Zhao et al (79) | 2019 | China | mean age: 40.4 years | a retrospective cross-sectional study | 189 | 132 | 57 | 22.20% | - | - | DST |
| Deng et al (159) | 2011 | China | mean age:40.1 ± 18.9 years | a retrospective cross-sectional study | 989 | 648 | 341 | 18.90% | - | - | DST |
| Wang et al (1999) (40) | 2012 | Zhejiang Province, China | 0–14, 15–64, >65 | cross sectional | 1013 | 723 | 290 | 13.60% | 13.80% | 13.10% | Drug sensitivity tests |
| Wang et al (2004) (40) | 2012 | Zhejiang Province, China | 0–14, 15–64, >65 | cross sectional | 984 | 730 | 254 | 16.20% | 17.10% | 13.40% | Drug sensitivity tests |
| Wang et al (2008) (40) | 2012 | Zhejiang Province, China | 0–14, 15–64, >65 | cross sectional | 938 | 646 | 292 | 13.30% | 14.10% | 11.60% | Drug sensitivity tests |
| El Achkar et al (83) | 2019 | Lebanon, | 34 ± 14 | cross sectional | 250 | 128 | 122 | 6.40% | - | - | Xpert testing |
| Lee et al (84) | 2009 | Korea | median age:21 yr | retrospective | 148 | 148 | 0 | 11.50% | - | - | drug susceptibility test |
| Buyankhishig et al (85) | 2011 | Mongolia | - | cross sectional | 850 | 507 | 343 | 18.20% | 64.50% | 35.50% | - |
| Seddon et al(86) | 2012 | Cape Town, South Africa | a median age of 26 months | cross sectional | 292 | 156 | 136 | 14% | - | - | DST |
| Alikhanova et al (90) | 2014 | Republic of Azerbaijan |  | cross sectional | 789 | - | - | 33.30% | - | - | A standard questionnaire |
| Tasbiti et al (91) | 2017 | Tehran, Iran | 46.6 ± 18.8 | a retrospective cross-sectional study | 1442 | **-** | **-** | 11.60% | - | - | Sputum culture and drug susceptibility testing (DST) |
| Cox et al (92) | 2004 | central Asia | mean age: 34 y, median age: 31 y | cross sectional survey | 213 | 60% | 40% | 53.10% | - | - | - |
| Otokunefor et al (93) | 2018 | Port Harcourt, Nigeria | - | cross sectional | 609 | 399 | 210 | 6.70% | - | - | DST |
| Wang et al (96) | 2016 | China | - | - | 2794 | 1937 | 857 | 15.70% | - | - | DST |
| Wang et al (97) | 2010 | China | - | - | 967 | 642 | 325 | 40.00% | - | - | - |
| Laghari et al (98) | 2019 | Pakistan | mean age was 12.84 ± 2.54 years | cross sectional | 50 | 32 | 18 | 60% | - | - | - |
| Afroz et al (99) | 2014 | Narshingdi, Bangladesh | range :25–34 years | cross sectional | 152 | 65 | 87 | 7.23% | - | - | - |
| Faridi et al (100) | 2018 | Aligarh Region |  |  | 514 |  |  | 6.03% | - | - | - |
| Gebeyehu et al (160) | 2001 | Arsi Zone, Ethiopia | a mean age of 37 years | cross sectional study | 195 | 99 | 96 | 2.56% | - | - | - |
| Rashedi et al(102) | 2015 | Republic of Azerbaijan | - | cross sectional | 32 | - | - | 72% | - | - | drug susceptibility |
| Rashedi et al (102) | 2015 | North-West of Iran | - | cross sectional | 48 | - | - | 4% | - | - | drug susceptibility |
| Madukaji et al (161) | 2021 | North Central of Nigeria | Mean age was 38.6 ± 13.4 years | cross sectional prospective study | 150 | 50 | 53 | 96% | - | - | - |
| Khunjeli et al (162) | 2014 | Nepal | - | retrospective study | 62 | - | - | 4.80% | - | - | - |
| Surucuoglu et al (105) | 2005 | Western Turkey | The median age of the patients was 45 ± 15.24 years for males and 44 ± 18.79 years for females. | cross sectional | 355 | 273 | 82 | 5.90% | 18% | 13% | drug susceptibility test |
| Bhat et al (106) | 2014 | Saharia tribe of central India | - | cross sectional | 475 |  |  | 5.05% | - | - | - |
| Kinjal et al (107) | 2019 | Jamnagar District | - | cross sectional | 1332 |  |  | 9.25% | - | - | DST, LPA |
| Jacobs et al (108) | 2019 | Brazil | 87.0% were between 20 and 59 years old | descriptive study | 1574 | 1111 | 463 | 21.70% | - | - | - |
| Dodd et al (109) | 2016 | - | - | mathematical model | 58000 |  |  | 6.90% | - | - | - |
| Abouyannis et al (110) | 2014 | Malawi | mean ages of 40.7 and 36.4 years | cross sectional | 1777 in total (1196 new & 581 retreatment) | 1777 | 0 | 5.85% in total (32% new & 11.4% retreatment) | - | - | - |
| Abdella et al (111) | 2015 | Jimma, Southwest Ethiopia | the mean age of 31.67 ± 10.02 y | cross sectional study | 79 | 48 | 31 | 51.40% | - | - | DST, socio demographic |
| Weyer et al (112) | 2007 | Africa | 0–64, +64 | cross sectional | 5866 | 3746 | 2115 | 7.40% | - | - |  |
| Rosas et al (114) | 2015 | Mexico | Mean age was 38.5 ± 13.9 | cross sectional | 214 | 146 | 68 | 17.90% | - | - | DST |
| Cox et al (64) | 2010 | Khayelitsha, South Africa | (new cases: 32 years & previously treated: 36 years) | cross sectional survey | 535 | 322 | 212 | (7.1% new & 9.7% previously treated) | - | - | DST |
| Hang et al (116) | 2013 | Hanoi, Viet Nam | median age: 38.6 years | - | 489 | 386 | 103 | 28.20% | - | - | DST, spoligotyping |
| Shibabaw et al (71) | 2020 | Amhara region, Ethiopia | The median age of the participants was 30 years | cross sectional | 211 | 133 | 78 | 88.50% | - | - | genotypic and phenotypic drug susceptibility testing (DST), GeneXpert MTB/RIF assay |
| Anwaierjiang et al (120) | 2021 | Southern Xinjiang, China | mean age of 50.4 (±19.3) years | cross sectional | 346 | 167 | 179 | 30.63% | - | - | phenotypic drug susceptibility testing, whole genome sequencing (WGS) |
| Dagne et al (124) | 2021 | Ethiopia | most frequent age-group was | A health facility-based cross-sectional study | 209 | 140 | 64 | 68.10% | - | - | - |
| Micheni et al (125) | 2021 | Southwestern Uganda | The median age of all patients was 36 years | cross sectional study | 283 | 207 | 76 | 11% | - | - | smear microscopy or Cepheid GeneXpert |
| Lai et al (127) | 2008 | Taiwan, China | - | retrospective analysis | 2625 |  |  | 15.40% | - | - | - |
| Lukoye et al (128) | 2013 | Uganda | median age :34.6 years | cross sectional | in total1537 in total (1397 new & 140 previously treated) | 1018 | 519 | (5% new & 23.3% previously treated) | - | - | Culture and drug susceptibility testing |
| Sanjeev et al (163) | 2018 | North India | median age: 34 years | an observational study | 1103 | 649 | 454 | 11% | - | - | DST |
| Abebe et al (132) | 2012 | southwestern Ethiopia | median age :26.0 (± 10.7) years | cross sectional | 136 | 78 | 58 | 13.20% | - | - | DST |
| Kim, SJ et al (164) | 1997 | Korea | - | cross sectional | 2486 | 1633 | 853 | 7.70% | 8.50% | 6.20% | DST |
| Yoshiyama et al (133) | 2001 | northern Thailand | - | cross sectional | 1077 | 748 | 329 | 13.20% | - | - | DST, Ziehl-Neelsen method |
| Dewan et al (134) | 2005 | Republic of Lithuania | The median age was 45years | - | 1163 in total (818 new & 345 previously treated) | 854 | 309 | (25.3% new & 66.4 % previously treated) | - | - | - |
| Tuberculosis Research Committee (165) | 2007 | Japan | a mean age of 61.1 ± 18.5 years, 61.0 ±  22.4 years | cross sectional | 3122 | 2211 | 911 | (2.8% new & 18.9% previously treated) | - | - | DST |
| Sidze et al (136) | 2014 | Central Region of Cameroon | mean age :33.9± 12.3 years | cross sectional | 665 | 390 | 275 | 4.70% | - | - | DST |
| Mekonnen et al (137) | 2014 | Amhara National Regional State, Ethiopia | mean age: 35.6 years | cross sectional | 606 | 363 | 243 | 2.50% | - | - | - |
| Castiñeiras et al (137) | 2006 | Castilla-León, Spain | - | cross sectional | 985 | - | - | 1.90% | - | - | DST |
| Xiang et al (143) | 2011 | Chongqing, China | mean age: 42.3 years | cross sectional | 1089 | 728 | 361 | 14% | - | - | DST |
| Yang et al (144) | 2015 | China | - | - | 1771 in total (1174 new & 597 retreated) | - | - | (17.3% new & 36.4% retreated) | - | - | - |
| Beltran et al (145) | 2011 | Sinaloa, Mexico | - | cross sectional | 671 | - | - | (1997-2002: 29.8%, 2003-2004: 27.3% ) | - | - | susceptibility test |

**Table S3:** Summary of Characteristics of Included Studies of Prevalence of Rifampcin Resistant-TB.

| Author | Year | Region | Age | Time Horizon of Methodology | Total sample size | Men sample size | Women sample size | Total Prevalence of Rifampin-resistant TB | Prevalence of Rifampin-resistant TB in men | Prevalence of Rifampin-resistant TB in women | Instrument |
| --- | --- | --- | --- | --- | --- | --- | --- | --- | --- | --- | --- |
| Ayaz et al (17) | 2012 | Karachi, Pakistan | >27 | cross sectional | 1004 | - | - | 0.20% | - | - | - |
| Yang et al (19) | 2014 | Northeast China | 15–44 | cross sectional | 205 | - | - | 1.50% | - | - | basis of clinical examination, CXR^[[8]](#footnote-8)^,sputum smear microscopy and sputum culture |
| Mahabeer et al (21) | 2016 | KwaZulu-Natal, South Africa | less than 5 years old | A retrospective descriptive analysis2012-2014 | 903 | - | - | 2.80% | - | - | automated Maycobacterial Growth Indicator Tube 960 system |
| Timire et al (12) | 2019 | Zimbabwe | 27–42, median age=34 | cross sectional | 1272 | 766 | 506 | 1.57% | - | - | survey questionnaire eliciting socio-demographic |
| Dorjee et al (166) | 2020 | Mountainous Districts of India, Himachal Pradesh | median age: 45 years | cross sectional | 378 | 337 | 41 | 18% | 20.50% | 31.50% | - |
| Singhal et al (23) | 2017 | Punjab state of India | median age:37 years | cross sectional | 557 | - | - | 7.70% | - | - | immunochromatographic test |
| Santos et al (26) | 2010 | Midwestern Brazil | 1 to 77 | cross sectional | 132 | 106 | 26 | 0.75% | - | - | - |
| Ghebremichael et al (29) | 2008 | Sweden | - | cross sectional | 400 | 199 | 201 | 14.25% | - | - | spoligotyping, molecular fingerprinting, restriction fragment length polymorphism(RFLP^[[9]](#footnote-9)^) |
| Pardini et al (31) | 2009 | Abkhazia (Georgia) | Median age:41 | cross sectional | 326 | 252 | 74 | 0.30% | - | - | Drug susceptibility testing (DST), molecular typing |
| Jiao et al (32) | 2015 | China | average age:6.8 | cross sectional | 100 | 57 | 43 | 30% | - | - | Drug susceptibility testing |
| Jiao et al (32) | 2015 | China | average age:16.9 | cross sectional | 159 | 83 | 76 | 23.27% | - | - | Drug susceptibility testing |
| Jiao et al (32) | 2015 | China | >18, average age:40.8 | cross sectional | 191 | 122 | 69 | 30.07% | - | - | Drug susceptibility testing |
| Chand et al (148) | 2000 | Pune, India | median age:36 years | cross sectional | 1120 | 130 | 62 | 15.10% | - | - | Ziehl Neelsen method |
| Ismail Et al (149) | 2018 | South Africa | ≥18 years old | cross sectional | 101422 |  |  | 4.6% (3-4.9%) | - | - | population proportionate, clustersampling design |
| Wang et al (38) | 2002 | Henan, China | - | cross sectional | 1372 | 973 | 399 | 29.88% | - | - | Standard drug susceptibility testing |
| Phyu et al (41) | 2005 | Yangon, Myanmar | new cases: 36.69 ± 13.2 previously treated: 39.3 ± 12.6 | cross sectional | 357 | 246 | 111 | 8.12% | - | - | Drug susceptibility testing |
| Mohajeri et al (167) | 2014 | West of Iran, Kermanshah | 44.2 ± 17.4 | cross sectional | 125 | 85 | 40 | 28% | - | - | Antimicrobial susceptibility testing (AST^[[10]](#footnote-10)^) |
| Liu et al (150) | 1998 | New Jersey | <25 ,25–44 ,45–64,>65 | cross sectional | 1926 | 1207 | 719 | 4.30% | 5.10% | 2.90% | drug susceptibility testing, completion of chemotherapy, initial drug regimen ,directly observed therapy |
| Shah et al (43) | 2018 | Mumbai, India | 3 months to <16 years | retrospective cross-sectional study | 196 |  | - | 83.60% | - | - | GeneXpert,LPA,drug susceptibility testing(DST) |
| Ignatova et al (44) | 2006 | Tula Region, Russia | Mean Age:35.9 years | cross sectional | 87 | 87 | - | 75.90% | 75.90% | - | drug susceptibility testing |
| Kamolwat et al (46) | 2021 | Thailand | 45–54 | cross sectional | 1501 | - | - | 2.13% | - | - | Microscopy examination, mycobacterial culture, DST |
| Yi Hu et al (47) | 2013 | Rural China | - | cross sectional | 380 | 267 | 113 | 0.52% | - | - | Species identification and drug susceptibility testing |
| Huo et al (151) | 2018 | China | - | cross sectional | 542 | 340 | 202 | 32.28% | - | - | - |
| Zhao et al (49) | 2014 | Hunan, China | - | cross sectional | 171 | 33 | 10 | 26.90% | - | - | drug susceptibility testing (DST), Spoligotyping |
| Bruchfeld et al (152) | 2002 | Addis Ababa, Ethiopia | Mean Age :(clustered:29.3 non clustered:31.1) | cross sectional | 121 | 78 | 43 | 2.48% | - | - | Drug susceptibility testing |
| Dinic et al (51) | 2012 | Nigeria |  | cross sectional | 224 | 130 | 94 | 6.69% | - | - | Multiplex-nested PCR and DNA sequencing of resistance genes |
| Agarwal et al (153) | 2010 | Malatya, Turkey | Median of 32 years | cross sectional | 397 | 228 | 159 | 6.29% | - | - | drug susceptibility testing |
| Djuretic et al (53) | 2015 | United Kindom | 15–44 years | cross sectional | 25217 | 14214 | 10049 | 1.35% | - | - | - |
| Fairlie et al (55) | 2011 | Johannesburg, South Africa | Median Age:78 | cross sectional | 148 |  |  | 1.40% | - | - | Drug susceptibility testing (DST |
| Sharaf Eldin et al (56) | 2011 | Sudan | < = 30 ,31-44,> = 45 | cross sectional | 235 | 175 | 60 | 13.40% | - | - | Spoligotyping, Drug susceptibility testing |
| Diallo et al (154) | 2017 | Chad | 12–70 years | cross sectional | 311 | 224 | 87 | (1.61% new & 3.8% previously treated) | - | - | Drug-susceptibility testing (DST) |
| Saldanha et al (61) | 2019 | Western India | Median age:41.5y | cross sectional | 200 | 134 | 66 | 2.50% | - | - | - |
| Huo et al 2005 (48) | 2019 | China | - | cross sectional | 273 | 45 | 228 | 28.20% | - | - | Conventional drug susceptibility testing and Mycobacterium species identification |
| Huo et al 2015 (48) | 2019 | China | - | cross sectional | 269 | 69 | 200 | 36.40% | - | - | Conventional drug susceptibility testing and Mycobacterium species identification |
| Diriba et al (63) | 2019 | Ethiopia | (97.9% age>15 years /2.1% age <15 years) | cross sectional | 329 | 243 | 86 | 6.70% | - | - | - |
| Cox et al new (64) | 2010 | Khayelitsha, South Africa | - | cross sectional | 271 | 155 | 116 | 4.50% | - | - | Drug susceptibility testing (DST) |
| Cox et al previously treated (64) | 2010 | Khayelitsha, South Africa | - | cross sectional | 264 | 167 | 96 | 11.20% | - | - | Drug susceptibility testing (DST) |
| Ullah et al (155) | 2016 | Pakistan | mean age: 39.7 ± 18.5 years | cross sectional | 2367 | 1238 | 1129 | 10.20% | 10.10% | 10.30% | Drug susceptibility testing |
| Ombura et al(156) | 2016 | Mombasa, Kenya | median age:30 | cross sectional | 258 | 174 | 84 | 0.38% | 0.40% | 0.00% | Demographic and clinical data collection |
| Mesfin et al (69) | 2018 | Addis Ababa, Ethiopia | average age:34.4y | cross sectional | 226 | 133 | 93 | 39.40% | - | - | - |
| Kigozi et al (70) | 2018 | Uganda | - | cross sectional | 97 | - | - | 7.21% | - | - | - |
| Shibabaw et al (71) | 2020 | Amhara region, Ethiopia | median age: 30 y | cross sectional | 211 | 133 | 78 | 97.60% | - | - | GeneXpert MTB/RIF assay, genotypic and phenotypic drug susceptibility testing (DST). |
| Gilad et al (72) | 2000 | southern Israel | mean age:52 ± 20 y | cross sectional | 249 | 138 | 111 | 11% | - | - | - |
| Ramzan et al (73) | 2004 | Asia | - | cross sectional | 47 |  |  | 3% | - | - | - |
| Um SJ et al (74) | 2011 | Korea | - | cross sectional | 373 |  |  | 10.70% | - | - | - |
| Diandé et al (75) | 2019 | Burkina Faso | 2–39, ≥40 | cross sectional | 1141 | 845 | 295 | 3.59% | - | - | Drug susceptibility testing |
| Becerril-Montes et al (157) | 2013 | Mexico/United states | 15-65 years | - | 139 | 82 | 57 | 17.27% | - | - | Drug susceptibility testing (DST) for first-line anti-TB drugs |
| Jaleta et al (168) | 2017 | northwest Ethiopia | mean age: 36.6 ± 15.8 years | a retrospective cross-sectional study | 1820 | 1078 | 742 | 15.80% | - | - | Xpert MTB/RIF assay, DST |
| Zhao et al (79) | 2019 | China | mean age: 40.4 years | a retrospective cross-sectional study | 189 | 132 | 57 | 10.10% | - | - | DST |
| Araya et al (169) | 2020 | Addis Ababa, Ethiopia | mean age: 40.3 ± 18.7 years | retrospective cross-sectional study | 12685 | 7040 | 5634 | 9.80% | - | 10.30% | Gene Xpert® testing |
| Deng et al (159) | 2011 | China | mean age:40.1 ± 18.9 years | retrospective cross-sectional study | 989 | 648 | 341 | 16.10% | - | - | DST |
| Wang et al (1999) (170) | 2012 | Zhejiang Province, China | 0–14, 15–64, >65 | cross sectional | 1013 | 723 | 290 | 11.50% | 11.20% | 12.10% | Drug sensitivity tests |
| Wang et al (2004) (170) | 2012 | Zhejiang Province, China | 0–14, 15–64, >65 | cross sectional | 984 | 730 | 254 | 9.60% | 10.00% | 8.30% | Drug sensitivity tests |
| Wang et al (2008) (170) | 2012 | Zhejiang Province, China | 0–14, 15–64 , >65 | cross sectional | 938 | 646 | 292 | 8.00% | 8.50% | 6.90% | Drug sensitivity tests |
| El Achkar et al (171) | 2019 | Lebanon, | 34 ± 14 | cross sectional | 250 | 128 | 122 | 2.80% | - | - | Xpert testing |
| Lee et al (84) | 2009 | Korea | median age:21 yrs | retrospective | 148 | 148 | 0 | 8.10% | - | - | drug susceptibility test |
| Adejumo et al (172) | 2018 | Lagos Nigeria | 25–44 years | A retrospective review | 2497 | 1373 | 1124 | 23.40% | - | - | Xpert MTB/RIF assay |
| Buyankhishig et al (85) | 2011 | Mongolia | - | cross sectional | 850 | 507 | 343 | 8.90% | 67.10% | 32.90% | - |
| Seddon et al (86) | 2012 | Cape Town, South Africa | a median age of 26 months | cross sectional | 292 | 156 | 136 | 1.40% | - | - | DST |
| Smith et al (89) | 2017 | Hlabisa subdistrict, KwaZulu-Natal | 25–34 years | cross sectional | 489 | 239 | 250 | 1.00% | - | - | Xpertw MTB/RIF testing |
| Alikhanova et al (90) | 2014 | Republic of Azerbaijan | - | cross sectional | 789 | - | - | 17.87% | - | - | A standard questionnaire |
| Tasbiti et al(91) | 2017 | Tehran, Iran | 46.6 ± 18.8 | retrospective cross-sectional study | 1442 | - | - | 12.20% | - | - | Sputum culture and drug susceptibility testing (DST) |
| Cox et al (92) | 2004 | Central Asia | median age: 31y | cross sectional survey | 213 | 60% | 40% | 26.80% | - | - | - |
| Otokunefor et al(93) | 2018 | Port Harcourt, Nigeria | - | cross sectional | 609 | 399 | 210 | 3.30% | - | - | DST |
| Wang et al (96) | 2016 | China | - | - | 2794 | 1937 | 857 | 6.60% | - | - | DST |
| Wang et al (97) | 2010 | China | - | - | 967 | 642 | 325 | 34.20% | - | - | - |
| Laghari et al (98) | 2019 | Pakistan | mean age was 12.84 ± 2.54 years | cross sectional | 50 | 32 | 18 | 66% | - | - | - |
| Afroz et al (99) | 2014 | Narshingdi, Bangladesh | range: 25–34 years | cross sectional | 152 | 65 | 87 | 4.60% | - | - | - |
| Faridi et al (100) | 2018 | Aligarh Region | - | - | 514 |  |  | 4.08% | - | - | - |
| Rashedi et al (102) | 2015 | Republic of Azerbaijan | - | cross sectional | 32 |  |  | 69% | - | - | drug susceptibility |
| Rashedi et al (102) | 2015 | North-West of Iran | - | cross sectional | 48 |  |  | 8% | - | - | drug susceptibility |
| Gebrehiwet et al (173) | 2019 | Afar, Ethiopia | ≤ 25,26-35,36-45≥ 46 | cross sectional | 384 | 189 | 195 | 4.30% | - | - | gene xpert tests |
| Bitet et al (174) | 2020 | Kagarko, Kaduna State, Nigeria |  | cross sectional | 182 | 130 | 52 | 2.70% | - | - | GeneXpert assay |
| Madukaji et al (175) | 2021 | North Central of Nigeria | Mean age was 38.6 ± 13.4 years | cross sectional prospective study | 150 | 50 | 53 | 98% | - | - | - |
| Surucuoglu et al (105) | 2005 | western Turkey | The median age of the patients was 45 ± 15.24 years for males and 44 ± 18.79 years for females. | cross sectional | 355 | 273 | 82 | 1.40% | 11% | 2.40% | drug susceptibility test |
| Awais et al (176) | 2018 | Hazara Division, Pakistan | ≤ 15, 16–30, 31–45, 46–60, 60–75 and 76–90 years | - | 635 | 147 | 150 | 6.29% | - | - | - |
| Bhat et al (106) | 2014 | Saharia tribe of central India | - | cross sectional | 475 |  |  | 0.84% | - | - | - |
| Kinjal et al (107) | 2019 | Jamnagar District | - | cross sectional | 1332 |  |  | 2.29% |  |  | DST, LPA |
| Jacobs et al (108) | 2019 | Brazil | 87.0% were between 20 and 59 years old | descriptive study | 1574 | 1111 | 463 | 9.00% | - | - | - |
| Abouyannis et al (110) | 2014 | Malawi | mean ages of 40.7 and 36.4 years | cross sectional | 1777 in total (1196 new & 581 retreatment) | 1777 | 0 | 2.64% in total (0.8% new & 6.5% retreatment) | - | - | - |
| Abdella et al (111) | 2015 | Jimma,Southwest Ethiopia | the mean age of 31.67 ± 10.02 y | cross sectional study | 79 | 48 | 31 | 32.90% | - | - | DST, socio demographic |
| Weyer et al (112) | 2007 | Africa | 0–64, +64 | cross sectional | 5866 | 3746 | 2115 | 3.40% | - | - | - |
| Soliman et al (113) | 2021 |  | - | cross sectional | 1069 |  |  | 27.20% | - | - | DST, gene xpert |
| Rosas et al (114) | 2015 | Mexico | Mean age was 38.5 ± 13.9 | cross sectional | 214 | 146 | 68 | 3.90% | - | - | DST |
| Cox et al (64) | 2010 | Khayelitsha, South Africa | (new cases: 32 years & previously treated : 36 years) | cross sectional survey | 535 | 322 | 212 | (4.5% new % 11.2% previously treated) | - | - | DST |
| Hang et al (116) | 2013 | Hanoi, Viet Nam | median age: 38.6 years | - | 489 | 386 | 103 | 4.90% | - | - | DST, spoligotyping |
| Shibabaw et al (71) | 2020 | Amhara region, Ethiopia | The median age of the participants was 30 years | cross sectional | 211 | 133 | 78 | 97% | - | - | genotypic and phenotypic drug susceptibility testing (DST), GeneXpert MTB/RIF assay |
| Bedaso et al (119) | 2021 | Bale Zone, Oromia Region,Ethiopia | - | A descriptive study | 43 | 14 | 29 | 69.80% | - | - | DST |
| Anwaierjiang et al (120) | 2021 | Southern Xinjiang, China | mean age of 50.4 (±19.3) years | cross sectional | 346 | 167 | 179 | 17.34% | - | - | phenotypic drug susceptibility testing, whole genome sequencing (WGS) |
| Patil et al (121) | 2019 | Western Maharashtra, India | - | prospective study | 1154 | - | - | 4.37% | - | - | GeneXpert MTB/RIF assay |
| Ikuabe et al (177) | 2018 | Yenagoa, Nigeria | - | A descriptive survey | 102 | - | - | 14.70% | - | - | Genexpert test |
| Dagne et al (178) | 2021 | Ethiopia | most frequent age-group was 25–34 years | A health facility-based cross-sectional study | 209 | 140 | 64 | 61.90% | - | - | - |
| Micheni et al (125) | 2021 | SouthWestern Uganda | The median age of all patients was 36 years | cross sectional study | 283 | 207 | 76 | 8.50% | - | - | smear microscopy or Cepheid GeneXpert |
| Lai et al (127) | 2008 | Taiwan, China | - | retrospective analysis | 2625 |  |  | 6.70% | - | - | - |
| Lukoye et al (128) | 2013 | Uganda | median age: 34.6 years | cross sectional | 1537 in total (1397 new & 140 previously treated ) | 1018 | 519 | (1.9% new & 12.1 % previously treated) | - | - | Culture and drug susceptibility testing |
| Fadeyi et al (179) | 2019 | North-Western, Nigeria | mean age: 35.9 ± 14.3 years | cross sectional | 120 | 73 | 47 | 4.20% | - | - | - |
| Masood et al (130) | 2019 | Karachi, Pakistan | - | A retrospective study | 315 |  |  | 27.90% | - | - | Chi-square test |
| Sanjeev et al (130) | 2018 | North India | median age :34 years | an observational study | 1103 | 649 | 454 | 9.10% | - | - | DST |
| Kim et al (164) | 1997 | Korea | - | cross sectional | 2486 | 1633 | 853 | 2.20% | 2.40% | 1.80% | DST |
| Yoshiyama et al (133) | 2001 | Northern Thailand | - | cross sectional | 1077 | 748 | 329 | 10.80% |  | -- | DST, Ziehl-Neelsen method |
| Dewan et al (134) | 2005 | Republic of Lithuania | The median age was 45years | - | 1163 in total (818 new & 345 previously treated) | 854 | 309 | (9.6% new & 54.2% previously treated) | - | - | - |
| Tuberculosis Research Committee (165) | 2007 | Japan | a mean age of 61.1 ± 18.5 years, 61.0 ±  22.4 years | cross sectional | 3122 | 2211 | 911 | (1% new & 11 % previously treated) | - | - | DST |
| Assiana et al (180) | 2021 | Brazzaville, Republic of Congo | < 18, 18–44≥45 | a cross-sectional study | 92 | 47 | 45 | 9.80% | - | - | DST |
| Sidze et al (136) | 2014 | Central Region of Cameroon | mean age :33.9± 12.3 years | cross sectional | 665 | 390 | 275 | 0.20% | - | - | DST |
| Mekonnen et al (137) | 2014 | Amhara National Regional State, Ethiopia | mean age: 35.6 years | cross sectional | 606 | 363 | 243 | 2.80% | - | - | - |
| Castiñeiras et al(138) | 2006 | Castilla-León, Spain | - | cross sectional | 985 | - | - | 0.30% | - | - | DST |
| Yang et al (144) | 2015 | China | - | - | 1771 in total (1174 new & 597 retreated) | - | - | (10.6% new & 28.9% retreated) | - | - | - |
| Beltran et al (145) | 2011 | Sinaloa, Mexico | - | cross sectional | 671 | - | - | (1997-2002: 19.2%, 2003-2004: 18.2%) | - | - | susceptibility test |

**Table S4:** Summary of Characteristics of Included Studies of Prevalence of Single Drug Resistant-TB.

| Author | Year | Region | Age | Type of study | Total sample size | Men sample size | Women sample size | Total Prevalence of single drug resistant TB | Prevalence of single drug resistant TB in men | Prevalence of single drug resistant TB in women | Instrument |
| --- | --- | --- | --- | --- | --- | --- | --- | --- | --- | --- | --- |
| Ahmad et al (13) | 2015 | Pakistan | 30.4 ± 14.4 | cross sectional | 289 | 108 | 135 | 3.40% | - | - | socio-demographic, microbiological, and clinical data |
| Ayaz et al (17) | 2012 | Karachi, Pakistan | >27 | cross sectional | 1004 | - | - | 14.60% | - | - | basis of clinical examination, CXR, sputum smear microscopy and sputum culture |
| Yang et al (19) | 2014 | Northeast China | 15–44 | cross sectional | 205 | - | - | 14.10% | - | - | - |
| Berberian et al (22) | 2016 | PediatríaGarrahan Argentina | 2–20 | Retrospective review 1998-2015 | 46 | - | - | 60% | - | - | - |
| Huo et al (181) | 2020 | China | <25,25–44,>64 | cross sectional | 542 | 74 | 37 | 20.40% | 66.70% | 33.30% | - |
| Ghebremichael et al(29) | 2008 | Sweden | - | cross sectional | 400 | 199 | 201 | 67.75% | - | - | spoligotyping, molecular fingerprintin, restriction fragment length polymorphism (RFLP) |
| Montoro et al (30) | 2006 | Cuba | - | cross sectional | 1528 |  |  | 7.20% | - | - | Drug susceptibility testing (DST) |
| Pardini et al (31) | 2009 | Abkhazia (Georgia) | Median age:41 | cross sectional | 326 | 252 | 74 | 23.31% | - | - | Drug susceptibility testing (DST), molecular typing |
| Van Rie et al (182) | 2000 | Cape Town, South Africa |  | cross sectional | 63 |  |  | 19.04% | - | - | RFLP |
| Chand et al (148) | 2000 | Pune, India | median age:36 years | cross sectional | 1120 | 130 | 62 | 8.84% | - | - | Ziehl Neelsen method |
| Daniel et al (37) | 2011 | Southwest, Nigeria | Men:35.9 ± 10.0 , Women:33.5 ± 9.3 | retrospective study | 88 | 50 | 38 | 12.70% | - | - | Drug susceptibility test |
| Wang et al (38) | 2002 | Henan, China |  | cross sectional | 1372 | 973 | 399 | 14.43% | - | - | Standard drug susceptibility testing |
| Phyu et al (41) | 2005 | Yangon, Myanmar | new cases: 36.69 ± 13.2 previously treated: 39.3 ± 12.6 | cross sectional | 357 | 246 | 111 | 12.88% | - | - | Drug susceptibility testing |
| Shah et al (43) | 2018 | Mumbai, India | 3 months to <16 years | retrospective cross-sectional study | 196 |  |  | 7.14% | - | - | GeneXpert,LPA,drug susceptibility testing(DST) |
| Bruchfeld et al (152) | 2002 | Addis Ababa, Ethiopia | Mean Age :(clustered:29.3 non clustered:31.1) | cross sectional | 121 | 78 | 43 | 9.09% | - | - | Drug susceptibility testing |
| Djuretic et al (53) | 2015 | United Kingdom | 15–44 years | cross sectional | 25217 | 14214 | 10049 | 5.16% | - | - | - |
| Isaakidis P et al (183) | 2014 | Metropolitan Mumbai, India | median age:35.0 | cross sectional | 1724 | 1042 | 671 | 21% | - | - | - |
| Mesfin et al (69) | 2018 | Addis Ababa, Ethiopia | average age:34.4y | cross sectional | 226 | 133 | 93 | 8.80% | - | - | - |
| Gilad et al (72) | 2000 | Southern Israel | mean age:52 ± 20 y | cross sectional | 249 | 238 | 111 | 13% | - | - | - |
| Becerril-Montes et al (157) | 2013 | Mexico/United states | 15-65 years | - | 139 | 82 | 57 | 20.86% | - | - | Drug susceptibility testing (DST) for first-line anti-TB drugs |
| Bojorquez-Chapela et al (87) | 2012 | Mexico | (42.9 ±47.7) | cross sectional | 2121 | 64.50% | 35.50% | 11.60% | - | - | DST |
| Surucuoglu et al (105) | 2005 | Western Turkey | The median age of the patients was 45±15.24 years for males and 44±18.79 years for females. | cross sectional | 355 | 273 | 82 | 16.30% | - | - | DST |
| Abdella et al (111) | 2015 | Jimma, Southwest Ethiopia | the mean age of 31.67 ± 10.02 y | cross sectional study | 79 | 48 | 31 | 14.30% | - | - | DST, socio demographic |
| Weyer et al (112) | 2007 | Africa | 0-64, +65 | cross sectional | 5866 | 3746 | 2115 | 5.10% | - | - | - |
| Rosas et al (114) | 2015 | Mexico | Mean age was 38.5 ± 13.9 | cross sectional | 214 | 146 | 68 | 15.60% | - | - | DST |
| Masood et al (130) | 2019 | Karachi, Pakistan | - | A retrospective study | 315 | - | - | 4.80% | - | - | Chi-square test |
| Castiñeiras et al (138) | 2006 | Castilla-León, Spain | - | cross sectional | 985 | - | - | 3.30% | - | - | DST |

**Table S5:** Summary of characteristics of included studies of prevalence of XDR-TB.

| Author | Year | Region | Age, years | Type of study | total sample size | Men sample size | Women sample size | Total prevalence of extensive drug resistant TB | Prevalence of extensive drug resistant TB in men | prevalence of extensive drug resistant TB in women | Instrument |
| --- | --- | --- | --- | --- | --- | --- | --- | --- | --- | --- | --- |
| Timire et al (184) | 2019 | Zimbabwe | 27-42, median age=34 | cross sectional | 1272 | 766 | 506 | 0.08% | - | - | survey questionnaire eliciting socio-demographic |
| Ahmad et al (13) | 2015 | Pakistan | 30.4 ± 14.4 | cross sectional | 289 | 108 | 135 | 4.49% | - | - | - |
| Hu et al (15) | 2015 | Rural China | 61 ± 15.3 | cross sectional | 1332 | 10 | 5 | 1.12% | 0.75% | 0.37% | Spoligotyping, DNA isolation and sequencing of loci |
| Mahabeer et al (21) | 2016 | KwaZulu-Natal, South Africa | less than 5 years old | A retrospective descriptive analysis (2012-2014) | 903 | - | - | 0-2.1% | - | - | automated Mycobacterial Growth Indicator Tube 960 system |
| Ghebremichael et al (185) | 2008 | Sweden | - | cross sectional | 400 | 199 | 201 | 0.25% | - | - | spoligotyping, molecular fingerprintin,restriction fragment length polymorphism(RFLP) |
| Pardini et al(31) | 2009 | Abkhazia (Georgia) | Median age:41 | cross sectional | 326 | 252 | 74 | 0.92% | - | - | Drug susceptibility testing (DST), molecular typing |
| Jiao et al (186) | 2015 | China | <15, average age:6.8 | cross sectional | 100 | 56+1 | 43 | 1% | - | - | Drug susceptibility testing |
| Jiao et al (186) | 2015 | China | average age:16.9 | cross sectional | 159 | 83 | 76 | 1.86% | - | - | Drug susceptibility testing |
| Jiao et al(186) | 2015 | China | average age:40.8 | cross sectional | 191 | 122 | 69 | 3.14% | - | - | Drug susceptibility testing |
| Brandao et al (187) | 2020 | Sao Paulo, Brazil | 37 ± 13 | cross sectional | 283 | 206 | 77 | 1.06% | - | - | Xpert tests, Phenotypic drug susceptibility testing, Gene sequencing,2.5. Molecular typing |
| Ismail Et al (149) | 2018 | south Africa | ≥18 years old | cross sectional | 101422 | - | - | 4.90% | - | - | population proportionate, cluster sampling design |
| Shah et al (43) | 2018 | Mumbai, India | 3 months to <16 years | retrospective cross-sectional study | 196 | - | - | 7.65% | - | - | GeneXpert,LPA,drug susceptibility testing(DST) |
| Kamolwat et al (46) | 2021 | Thailand | 45–54 | cross sectional | 1501 | - | - | 0.06% | - | - | Microscopy examination, mycobacterial culturists |
| Ahmad et al (188) | 2013 | Pakistan | - | cross sectional | 102 |  |  | 57% | - | - | Drug susceptibility testing, |
| Huo et al (151) | 2018 | China | - | cross sectional | 542 | 340 | 202 | 5.71% | - | - |  |
| Zhao et al (49) | 2014 | Hunan, China | - | cross sectional | 171 | 33 | 10 | 1.75% | - | - | drug susceptibility testing (DST), Spoligotyping |
| Daum et al (50) | 2018 | Ukraine | Mean Age:39 | cross sectional | 75 | 63 | 12 | 34.66% | - | - | DST |
| Xin-Tong et al (54) | 2017 | Dalian, China | 0–30, 31–59 ≥ 60 | cross sectional | 3522 in totalin total (2261 new&1291 previously treated) | 2680 | 872 | 2.13% in totalin total (1.10% new& 3.87% previously treated) | - | - | DST |
| Pang et al (146) | 2017 | Southern China | average:46.6y | cross sectional | 133 | 80 | 53 | 12.80% | - | - | Drug susceptibility testing |
| Cox et al new (64) | 2010 | Khayelitsha, South Africa | - | cross sectional | 271 | 155 | 116 | 0.40% | - | - | Drug susceptibility testing (DST) |
| Cox et al (64) previously treated | 2010 | Khayelitsha, South Africa | - | cross sectional | 264 | 167 | 96 | 0.50% | - | - | Drug susceptibility testing (DST) |
| Porwal et al (66) | 2013 | Delhi Region | 34.4 ± 8.9 | cross sectional | 611 | 12 | 6 | 2.94% | - | - | DST |
| Isaakidis et al(183) | 2014 | Metropolitan Mumbai, India | median age:35.0 | cross sectional | 1724 | 1042 | 671 | 6% | - | - | - |
| Um SJ et al (74) | 2011 | Korea | - | cross sectional | 373 |  |  | 1.00% | - | - | - |
| Zhao et al (79) | 2019 | China | mean age: 40.4 years | a retrospective cross-sectional study | 189 | 132 | 57 | 1.10% | - | - | DST |
| Migliori et al (80) | 2007 | Italy & Germany | - | cross sectional | 2888 in totalin total (2140 Italy & 748 Germany ) | - | - | 0.4% in total (0.37% Italy & 0.40% Germany) | - | - | - |
| Deng et al (159) | 2011 | China | mean age:40.1 ± 18.9 years | a retrospective cross-sectional study | 989 | 648 | 341 | 2.02% | - | - | DST |
| Wallengren et al (82) | 2011 | KwaZulu-Natal, South Africa | - | a retrospective cross-sectional study | 119218 | - | - | 9.60% | - | - | drug-sensitivity testing |
| El Achkar et al (83) | 2019 | Lebanon, | 34 ± 14 | cross sectional | 250 | 128 | 122 | 1.20% | - | - | Xpert testing |
| P. W. E I et al (88) | 2017 | Myanmar | - | a cross-sectional, retrospective study | 94 | - | - | 13.50% | - | - | DST |
| Smith et al (89) | 2017 | Hlabisa subdistrict, KwaZulu-Natal | 25–34 years | cross sectional | 489 | 239 | 250 | 1.20% | - | - | Xpertw MTB/FIR testing |
| Alikhanova et al (90) | 2014 | Republic of Azerbaijan | - | cross sectional | 789 | - | - | 13.04% | - | - | A standard questionnaire |
| Tasbiti et al (91) | 2017 | Tehran, Iran | 46.6 ± 18.8 | a retrospective cross-sectional study | 1442 | - | - | 0.20% | - | - | Sputum culture and drug susceptibility testing (DST) |
| Roshdi Maleki et al (94) | 2012 | East Azerbaijan province of Iran | - | cross sectional | 230 | 230 | 0 | 1.30% | - | - | Drug susceptibility testing |
| Wang et al (96) | 2016 | China | - | - | 2794 | 1937 | 857 | 0.40% | - | - | Drug susceptibility testing |
| Senthil Kumar et al (189) | 2020 | South Tamil Nadu | - | retrospective cross-sectional study | 173 | - | - | 2% | - | - |  |
| Chuchottaworn et al (190) | 2010 | Thailand | - | a retrospective review | 10289 | - | - | 0.37% | - | - | Drug susceptibility testing |
| Dodd et al(109) | 2016 |  | - | mathematical model | 58000 | - | - | 4.70% | - | - |  |
| Cox et al (64) | 2010 | Khayelitsha, South Africa | new cases: 32 years & previously treated : 36 years) | cross sectional survey | 535 | 322 | 212 | (0.4 % new % 0.5% previously treated) | - | - | DST |
| Minion et al (117) | 2013 | Canada | - | cross sectional | 15993 | 8784 | 7209 | 2.80% | - | - | DST |
| Agumas et al (71) | 2020 | Amhara region, Ethiopia | The median age of the participants was 30 years | cross sectional | 211 | 133 | 78 | 0.60% | - | - | genotypic and phenotypic drug susceptibility testing (DST),GeneXpert MTB/RIF assay |
| Gallo et al (118) | 2017 | Sao Paulo, Brazil | mean age: 40.2 ± 11.4years | cross sectional | 313 | 162 | 59 | 10.20% | 50.00% | 50.00% | DST |
| Adwani et al (123) | 2016 | Mumbai, India | - | - | 227 | 113 | 114 | 4.85% | 3.53% | 6.14% | DST |
| Dagne et al (124) | 2021 | Ethiopia | most frequent age-group was 25–34 years | A health facility-based cross-sectional study | 209 | 140 | 64 | 0.60% | - | - | - |
| Anees et al (126) | 2019 | Peshawar, Khyber PakhtonKhwa province of Pakistan | Mean age of all patients were 29.80±16.32 years. | cross sectional | 102 | - | - | 2.94% | 23.85% | 25.86% | gene expert procedure |
| Masood et al t(130) | 2019 | Karachi. Pakistan | - | A retrospective study | 315 | - | - | 1.60% | - | - | Chi-square test |
| Wang et al (142) | 2021 | China | - | - | 391 | - | - | 7.16% | - | - | DST |
| Yang et al (144) | 2015 | China | - | - | 1771 in total (1174 new & 597 retreated) | - | - | 12% | - | - | - |
| Yu Pang et al (191) | 2017 | China | median age: 42 years | A retrospective study | 9544 | 16 | 13 | 0.64% | - | - | DST |

**Table S6:** Summary of characteristics of included studies of prevalence of pre-XDR TB.

| (11) Author | Year | Region | Age | Type of study | Total sample size | Men sample size | Women sample size | Total prevalence of pre-XDR TB | prevalence of pre-XDR TB in Men | prevalence of pre-XDR TB in Women | Instrument |
| --- | --- | --- | --- | --- | --- | --- | --- | --- | --- | --- | --- |
| Hu et al (15) | 2015 | rural China | 55 ± 17.1 | cross sectional | 1332 | 28 | 17 | 3.37% | 2.10% | 1.27% | Spoligotyping, DNA isolation and sequencing of loci |
| Jiao et al (186) | 2015 | China | average age:6.8 | cross sectional | 100 | 57 | 43 | 8% | - | - | Drug susceptibility testing |
| Jiao et al (186) | 2015 | China | average age:16.9 | cross sectional | 159 | 83 | 76 | 13% | - | - | Drug susceptibility testing |
| Jiao et al (186) | 2015 | China | average age:40.8 | cross sectional | 191 | 122 | 69 | 23% | - | - | Drug susceptibility testing |
| Brandao et al (33) | 2020 | Sao Paulo, Brazil | 37 ± 13 | cross sectional | 283 | 206 | 77 | 3.18% | - | - | Xpert tests, Phenotypic drug susceptibility testing,Gene sequencing,2.5. Molecular typing |
| Shah et al (43) | 2018 | Mumbai, India | 3 months to <16 years | retrospective cross-sectional study | 196 | - | - | 30.60% | - | - | GeneXpert,LPA,drug susceptibility testing(DST) |
| Kamolwat et al (46) | 2021 | Thailand | 45–54 | cross sectional | 1501 | - | - | 0.13% | - | - | Microscopy examination, mycobacterial culture,DST |
| Ahmedet al (188) | 2013 | Pakistan | - | cross sectional | 102 |  |  | 42.10% | - | - | Drug susceptibility testing, |
| Zhao et al (49) | 2014 | Hunan, China | - | cross sectional | 171 | 33 | 10 | 8.70% | - | - | drug susceptibility testing (DST), Spoligotyping |
| Pang et al (146) | 2017 | Southern China | average:46.6y | cross sectional | 133 | 80 | 53 | 28.60% | - | - | Drug susceptibility testing |
| Porwal et al (66) | 2013 | Delhi Region | 32 ± 12.81 | cross sectional | 611 | 25 | 28 | 8.67% | - | - | DST |
| Isaakidis et al (67) | 2014 | Metropolitan Mumbai, India | median age:35.0 | cross sectional | 1724 | 1042 | 671 | 21% | - | - | - |
| Zhao et al (79) | 2019 | China | mean age: 40.4 years | retrospective cross-sectional study | 189 | 132 | 57 | 3.20% | - | - | DST |
| P. W. E I et al (88) | 2017 | Myanmar | - | a cross-sectional, retrospective study | 94 | - | - | 27% | - | - | DST |
| Alikhanova et al (90) | 2014 | Republic of Azerbaijan | - | cross sectional | 789 | - | - | 28.26% | - | - | A standard questionnaire |
| Diriba et al (192) | 2022 | Ethiopia | mean age: 29 ± 11.8 years. | A laboratory-based cross-sectional study | 644 | 400 | 244 | 3% | - | - | drug-susceptibility testing (DST), sociodemographics |
| Senthil Kumar et al (189) | 2020 | South Tamil Nadu | - | retrospective cross-sectional study | 173 |  |  | 19% | - | - | - |
| Agumas et al (71) | 2020 | Amhara region, Ethiopia | The median age of the participants was 30 years | cross sectional | 211 | 133 | 78 | 5.70% | - | - | genotypic and phenotypic drug susceptibility testing (DST),GeneXpert MTB/RIF assay |
| Gallo et al(118) | 2017 | S~ao Paulo, Brazil | mean age :39.0 ± 12.1 years | cross sectional | 313 | 162 | 59 | 19.20% | 70% | 30% | DST |
| Adwani et al (123) | 2016 | Mumbai, India | - | - | 227 | 113 | 114 | 55.95% | 61.90% | 50% | DST |
| Dagne et al (124) | 2021 | Ethiopia | most frequent age-group was 25–34 years | A health facility-based cross-sectional study | 209 | 140 | 64 | 5% | - | - | - |
| Assiana et al (180) | 2021 | Brazzaville, Republic of Congo | < 18, 18-44 ≥45 | cross sectional study | 92 | 47 | 45 | 2.20% | - | - | DST |
| Wang et al (142) | 2021 | China | - | - | 391 |  |  | 24.04% | - | - | DST |

DST: drug susceptibility testing

1. Drug susceptibility testing [↑](#footnote-ref-1)
2. Line probe assay [↑](#footnote-ref-2)
3. Polymerase chain reaction [↑](#footnote-ref-3)
4. Pyrazinamide susceptibility testing [↑](#footnote-ref-4)
5. Mycobacterium tuberculosis [↑](#footnote-ref-5)
6. Rifampcin [↑](#footnote-ref-6)
7. Whole genome sequencing [↑](#footnote-ref-7)
8. Chest X-ray [↑](#footnote-ref-8)
9. restriction fragment length polymorphism [↑](#footnote-ref-9)
10. Antimicrobial susceptibility testing [↑](#footnote-ref-10)
