# Supplementary material for: Global prevalence of drug-resistant tuberculosis: a systematic review and meta-analysis
Source: Infect Dis Poverty. 2023 May 25;12:57. doi: 10.1186/s40249-023-01107-x (PMC10210422; doi:10.1186/s40249-023-01107-x)
Supplement: Supplementary file 2 — Additional file 2: Figure S1. Forest plot of the global prevalence ofmulti-drug resistant TB based on the random effects method. Figure S2. Funnel plotof publication bias in reviewed studies. Figure S3.Forest plot of global prevalence of isoniazid resistant TB based on randomeffects method. Figure S4. Funnel plot of publication biasin reviewed studies. Figure S5. Forest plot ofglobal prevalence of rifampin-resistant TB based on random effects method. FigureS6.Funnel plot of publication bias in reviewed studies. FigureS7. Forest plot of globalprevalence of single drug resistant TB based on random effects method. FigureS8.Funnel plot of publication bias in reviewed studies.Figure S9. Forest plot of global prevalence of extensively drugresistant TB based on random effects method. Figure S10. Funnel Plot of Publication Bias in ReviewedStudies. [file 40249_2023_1107_MOESM2_ESM.docx]

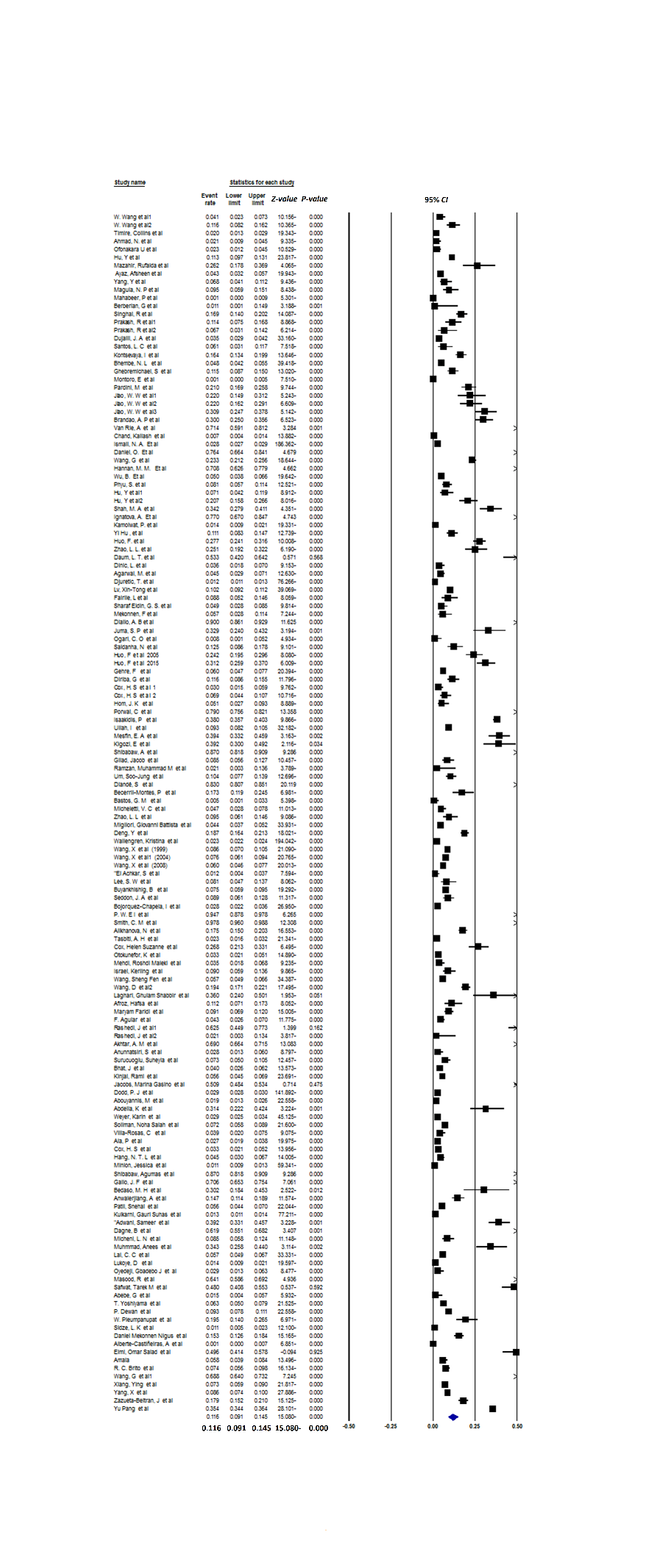


**Figure S1:** Forest plot of the global prevalence of multi-drug resistant TB based on the random effects method.

**
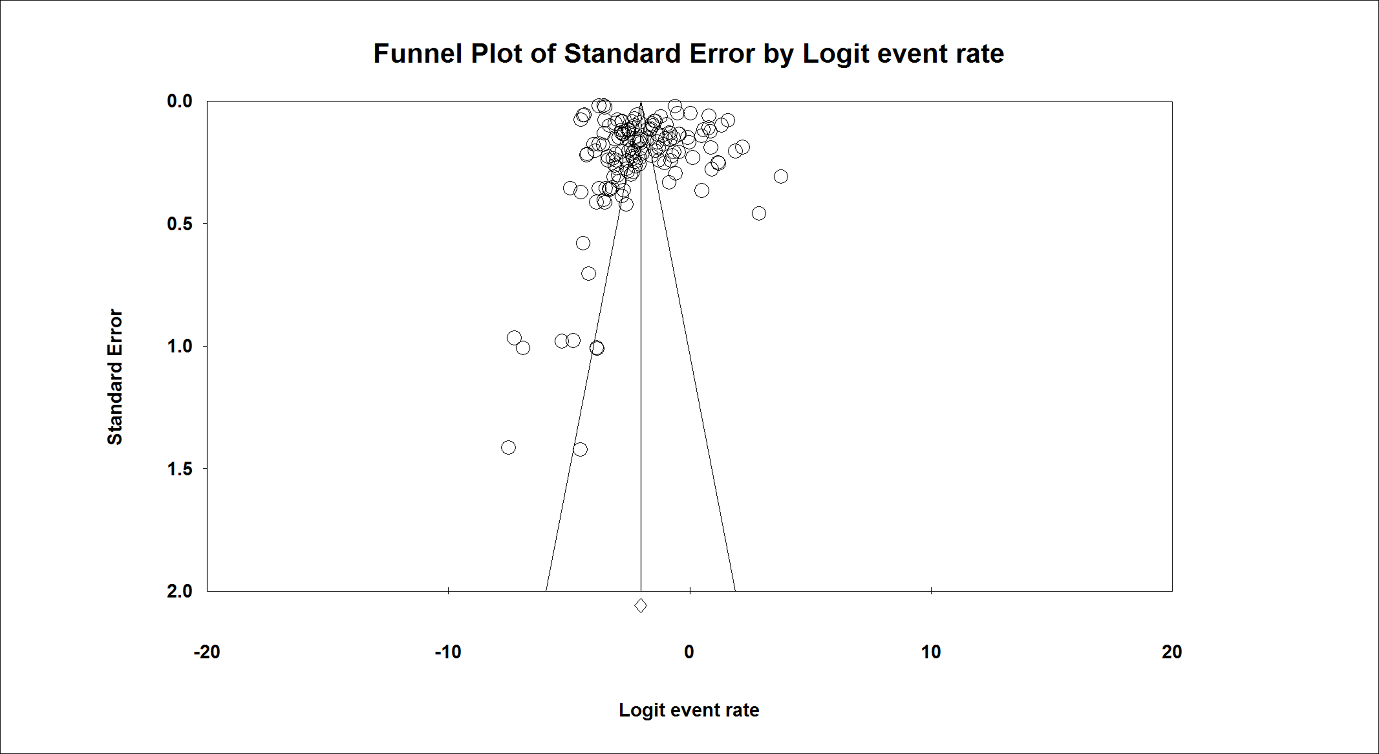
Figure S2:** Funnel plot of publication bias in reviewed studies (multi-drug resistant TB).


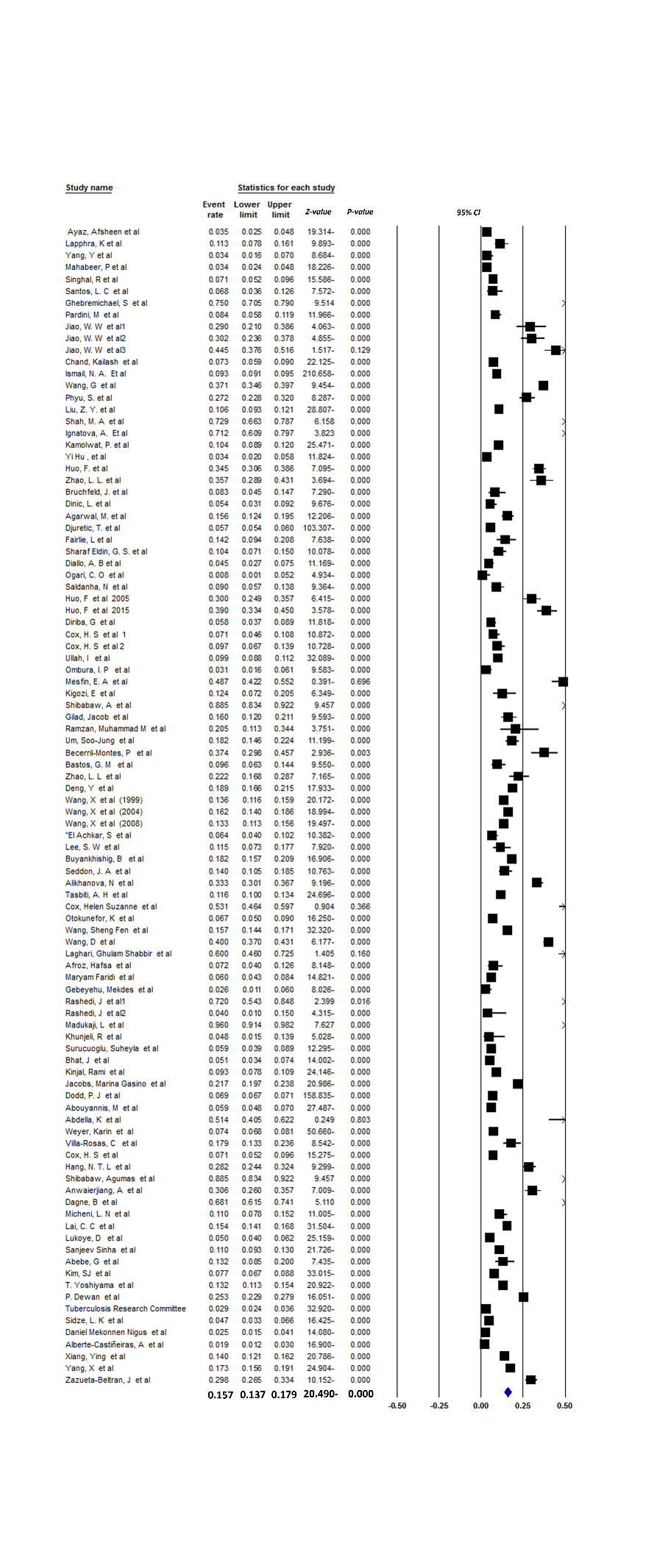


**Figure S3:** Forest plot of global prevalence of isoniazid resistant TB based on random effects method.


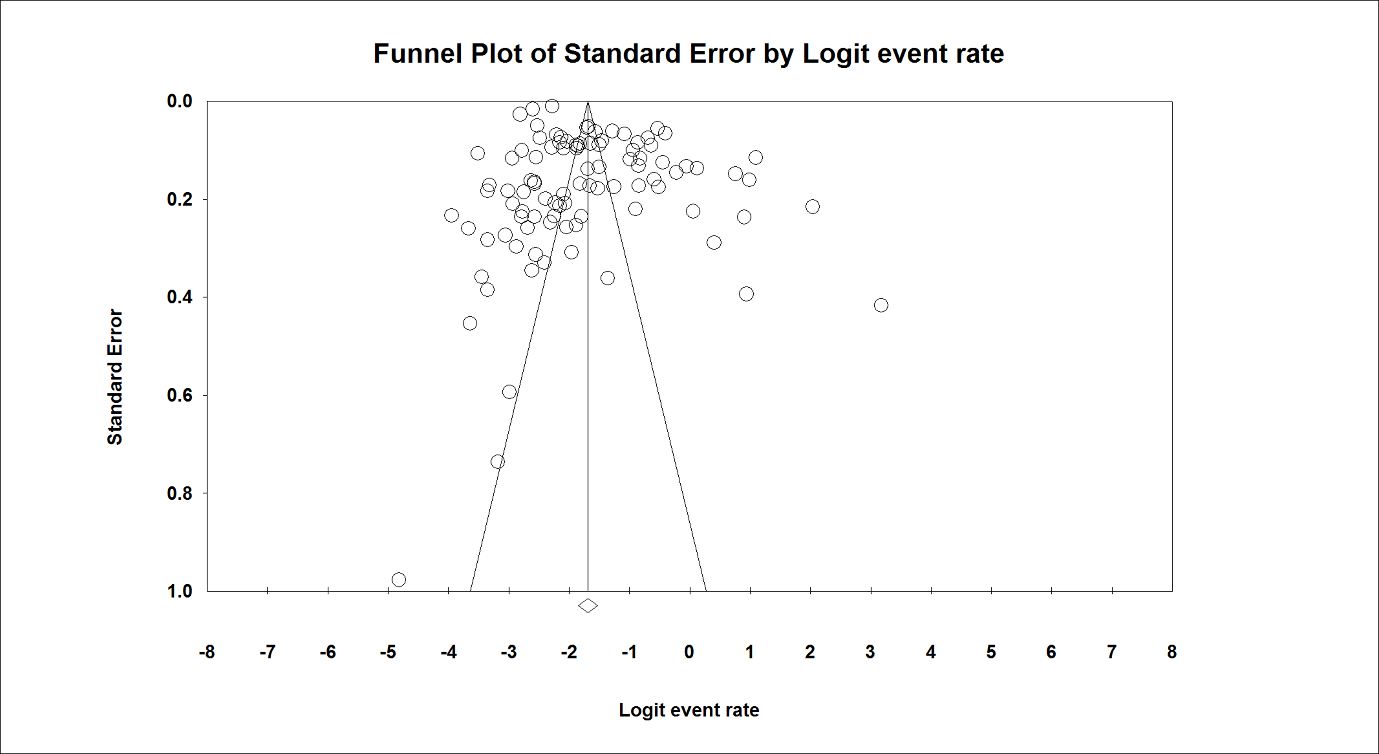


**Figure S4:** Funnel plot of publication bias in reviewed studies (isoniazid resistant TB).


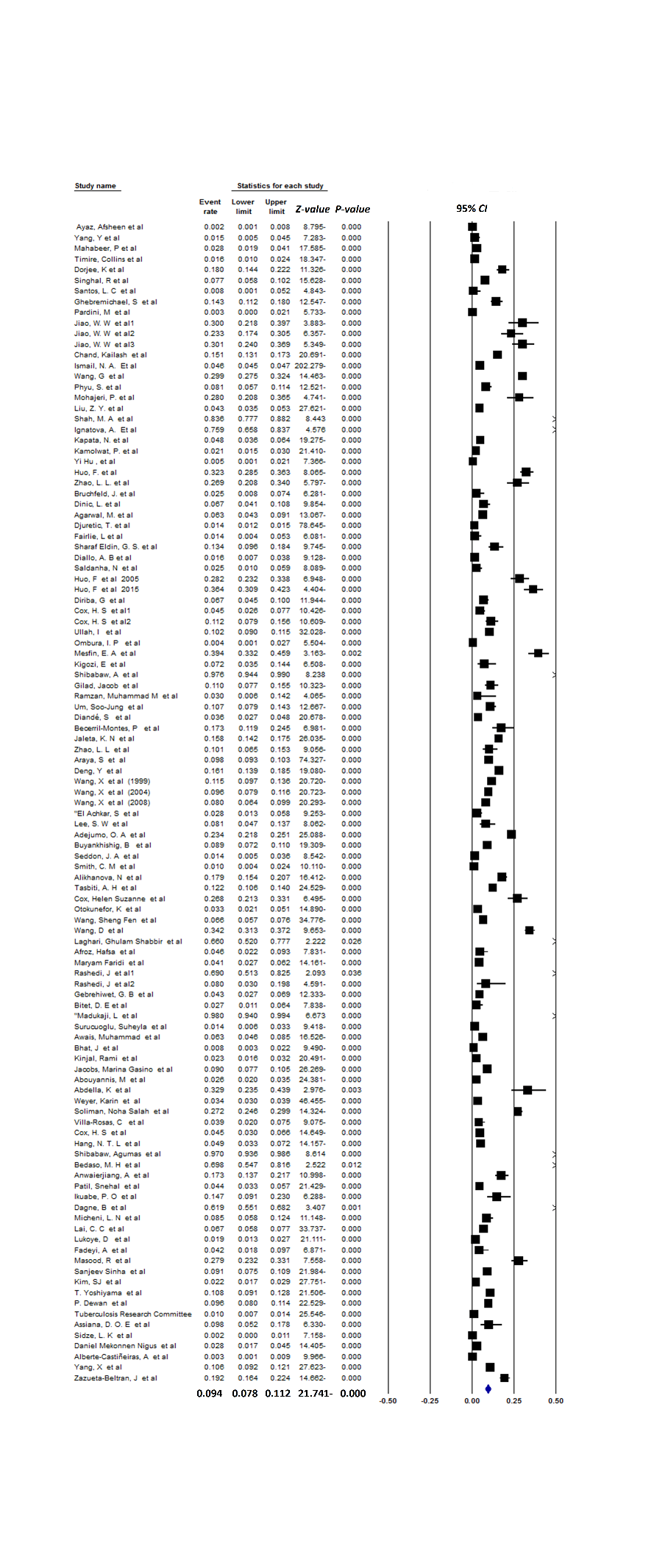


**Figure S5:** Forest plot of global prevalence of rifampin-resistant TB based on random effects method.

**
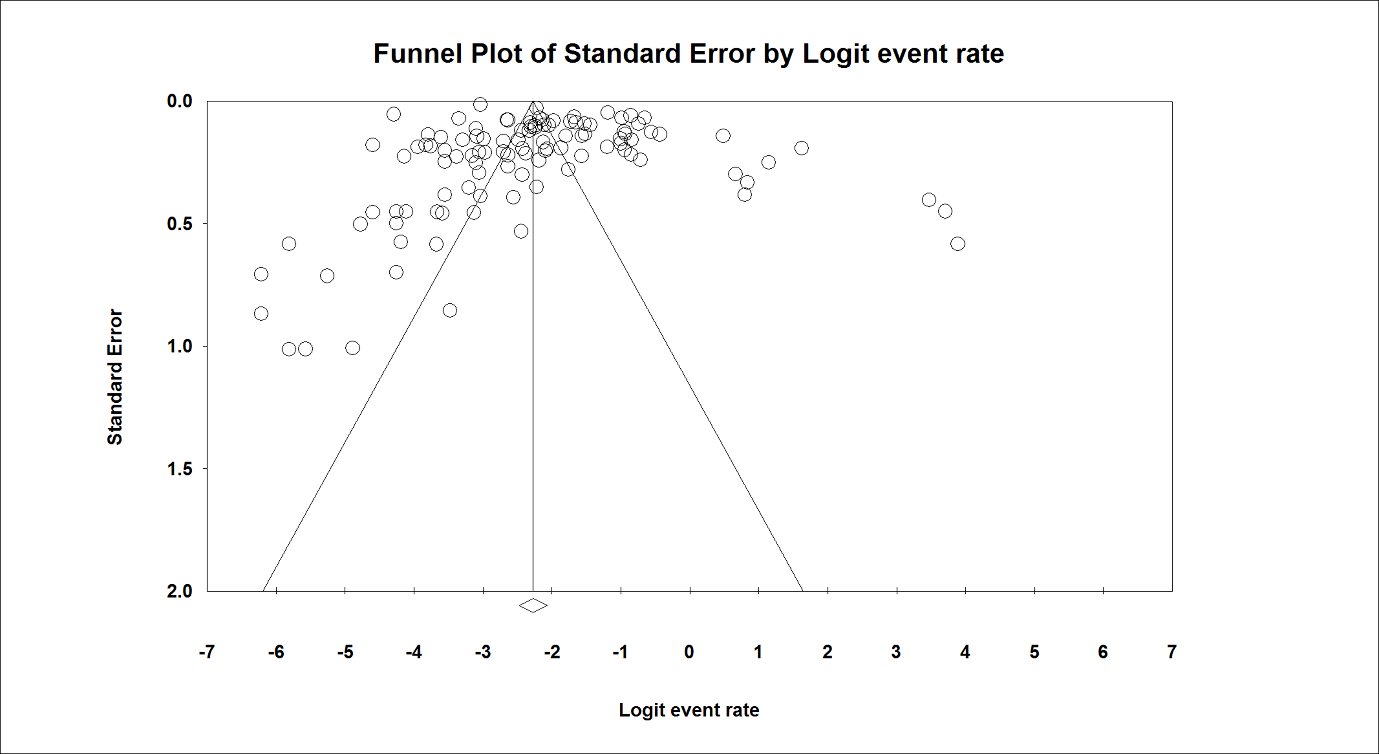
Figure S6:** Funnel plot of publication bias in reviewed studies (rifampin-resistant TB).


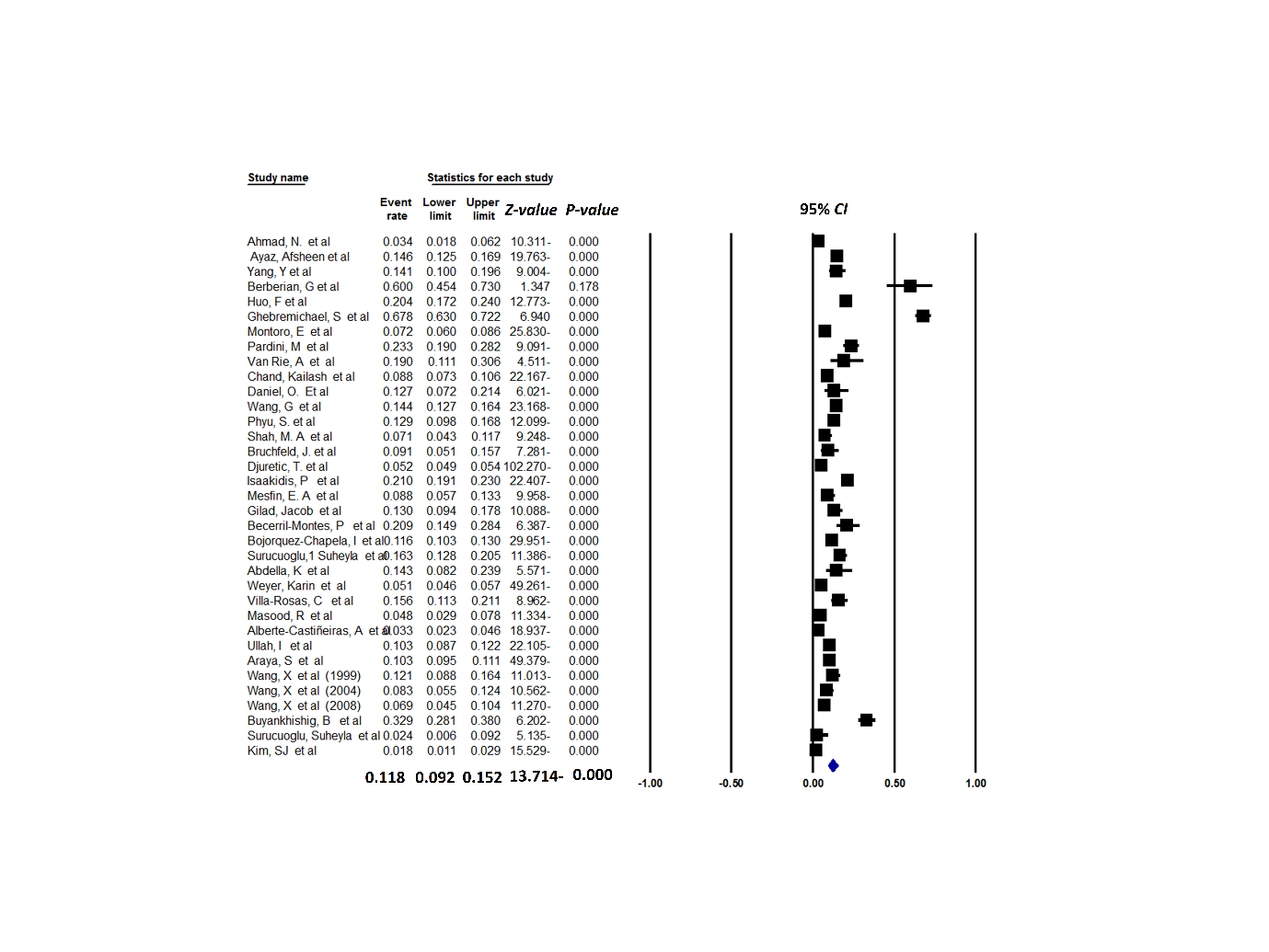


**Figure S7:** Forest plot of global prevalence of single drug resistant TB based on random effects method.

**
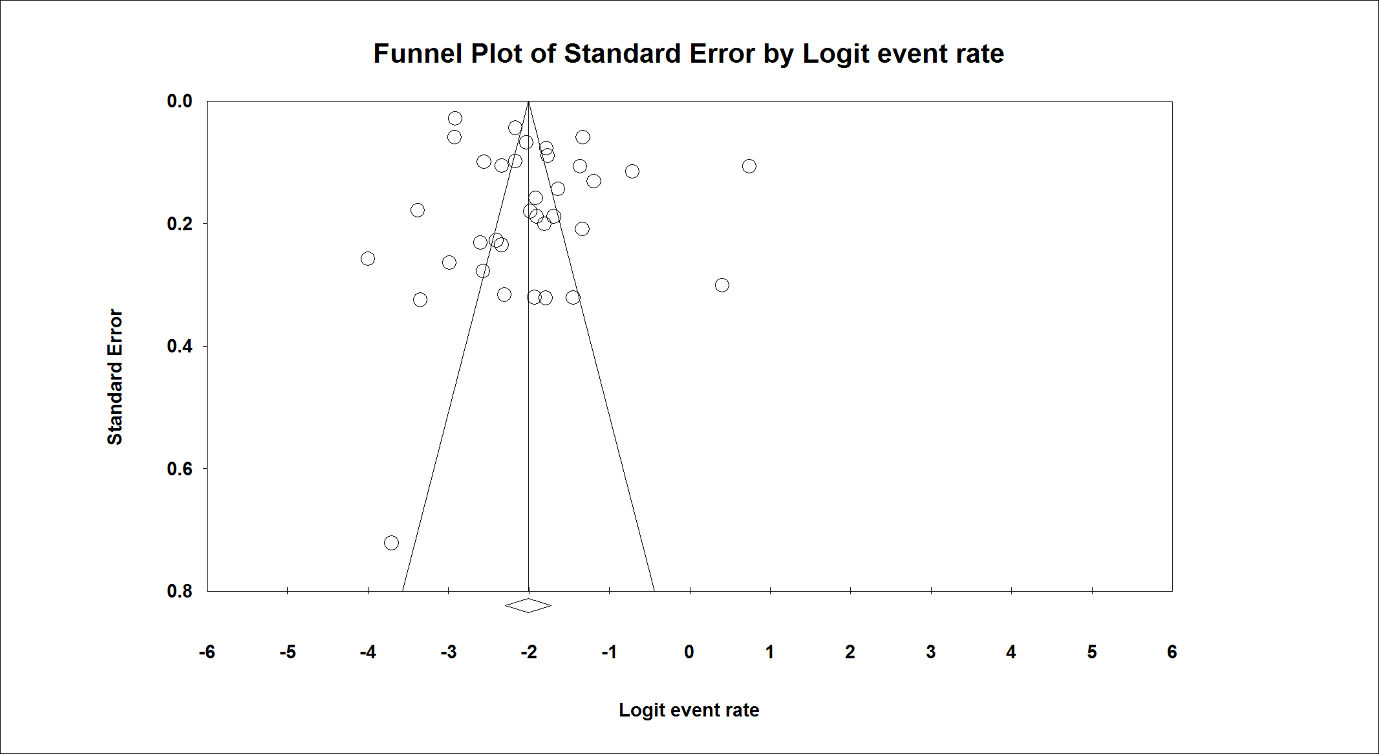
Figure S8:** Funnel plot of publication bias in reviewed studies (single drug resistant TB).

**
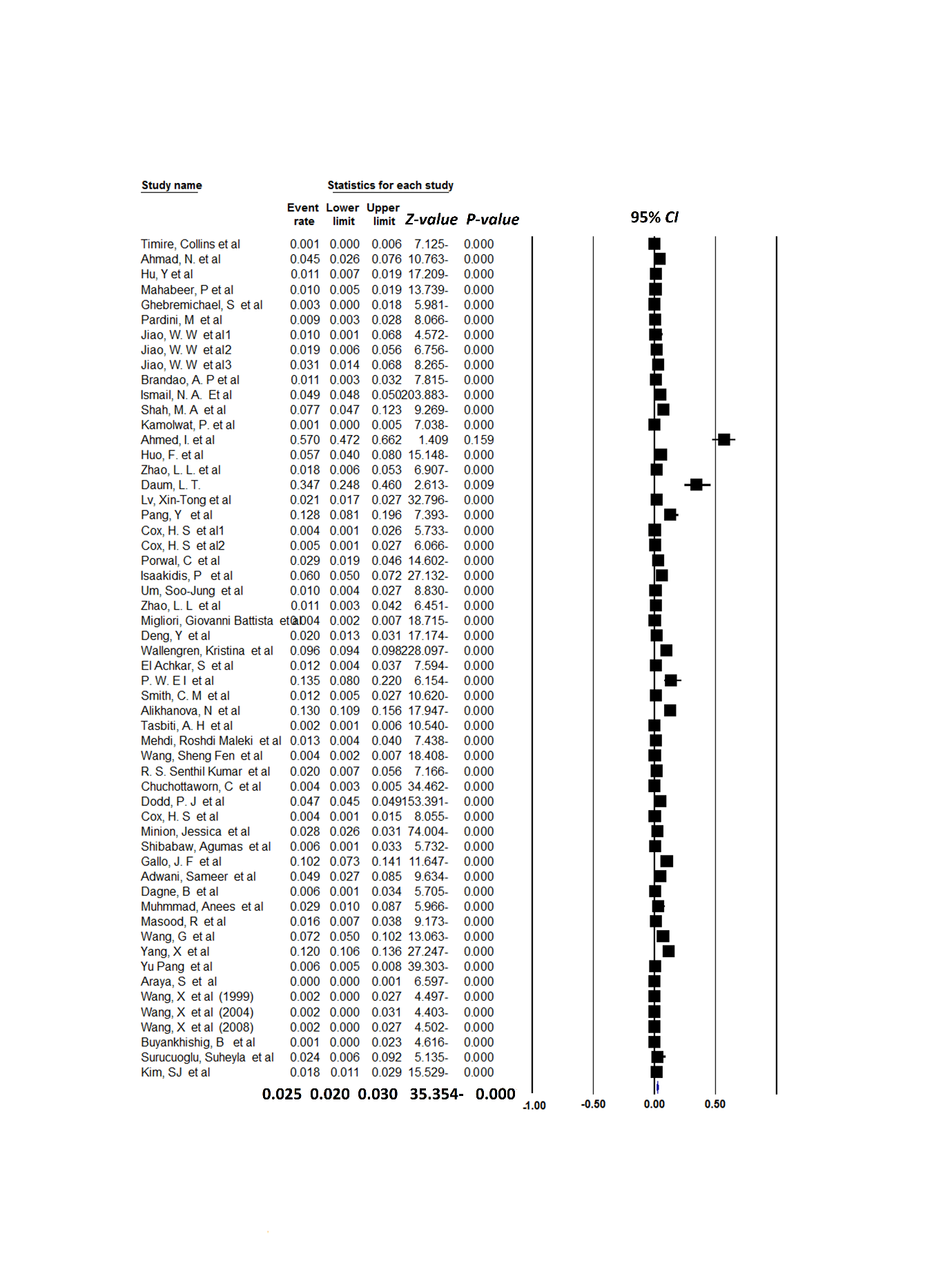
Figure S9:** Forest plot of global prevalence of extensively drug resistant TB based on random effects method.

**
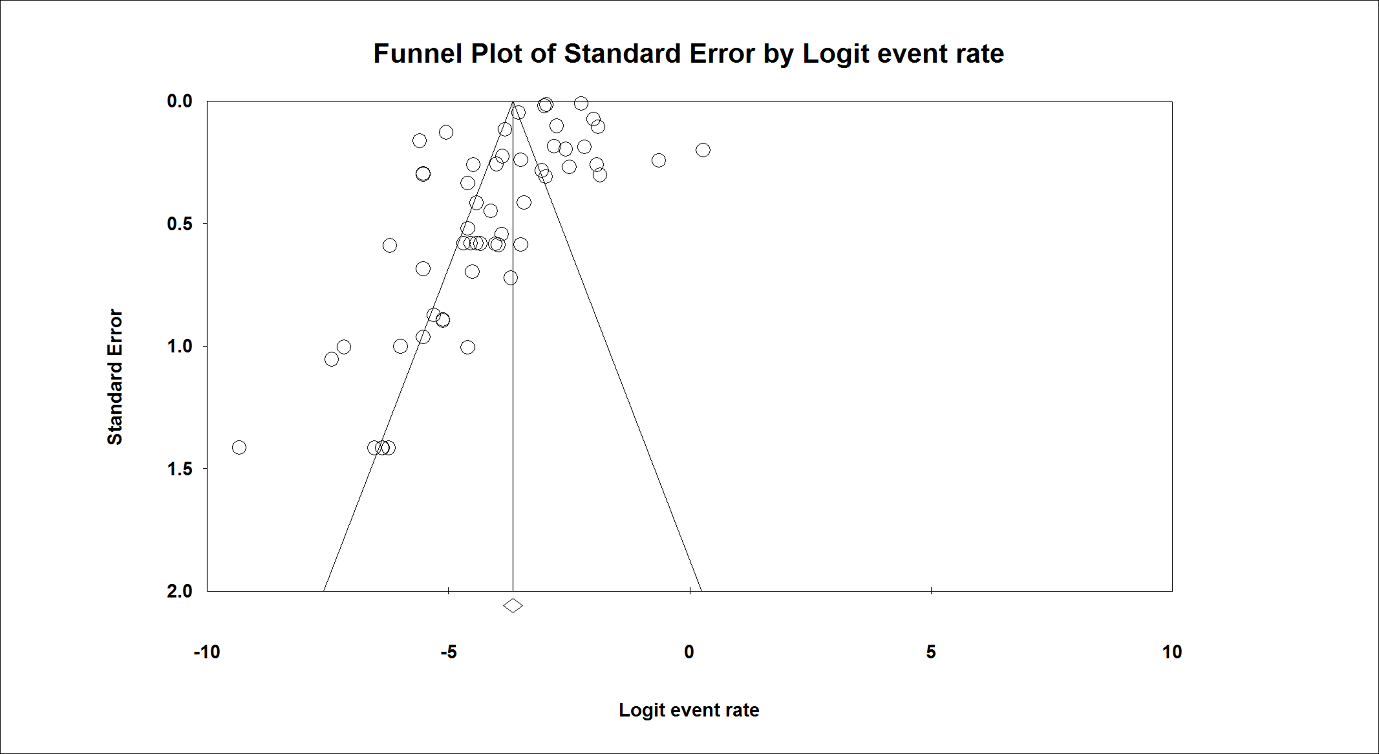
Figure S10:** Funnel Plot of Publication Bias in Reviewed Studies (Extensively Drug Resistant TB).
